# Abnormal global signal topography of self modulates emotion dysregulation in major depressive disorder

**DOI:** 10.1038/s41398-023-02398-2

**Published:** 2023-04-03

**Authors:** Kaan Keskin, Mehmet Çağdaş Eker, Ali Saffet Gönül, Georg Northoff

**Affiliations:** 1 Department of Psychiatry, Ege University, Izmir, Turkey; 2 SoCAT Lab, Ege University, Izmir, Turkey; 3 Mind, Brain Imaging and Neuroethics Research Unit, University of Ottawa, Ontario, ON Canada

**Keywords:** Depression, Neuroscience

## Abstract

Major depressive disorder (MDD) is a complex mental disorder featured by an increased focus on the self and emotion dysregulation whose interaction remains unclear, though. At the same time, various studies observed abnormal representation of global fMRI brain activity in specifically those regions, e.g., cortical midline structure (CMS) in MDD that are associated with the self. Are the self and its impact on emotion regulation related to global brain activity unevenly represented in CMS relative to non-CMS? Addressing this yet open question is the main goal of our study. We here investigate post-acute treatment responder MDD and healthy controls in fMRI during an emotion task involving both attention and reappraisal of negative and neutral stimuli. We first demonstrate abnormal emotion regulation with increased negative emotion severity on the behavioral level. Next, focusing on a recently established three-layer topography of self, we show increased representation of global fMRI brain activity in specifically those regions mediating the mental (CMS) and exteroceptive (Right temporo-parietal junction and mPFC) self in post-acute MDD during the emotion task. Applying a complex statistical model, namely multinomial regression analyses, we show that increased global infra-slow neural activity in the regions of the mental and exteroceptive self modulates the behavioral measures of specifically negative emotion regulation (emotion attention and reappraisal/suppression). Together, we demonstrate increased representation of global brain activity in regions of the mental and exteroceptive self, including their modulation of negative emotion dysregulation in specifically the infra-slow frequency range (0.01 to 0.1 Hz) of post-acute MDD. These findings support the assumption that the global infra-slow neural basis of the increased self-focus in MDD may take on the role as basic disturbance in that it generates the abnormal regulation of negative emotions.

## Introduction

### Major depressive disorder—from the increased self-focus to emotion dysregulation

Major depressive disorder (MDD) is a complex mental disorder featured by the co-occurrence of affective, cognitive, social, somatic, and motor symptoms [1–3]. Such symptom coupling suggests a more global change in the organization of the relationship between these different symptoms/functions (rather than a specific local change in one particular function) [4]. One such feature providing a more global organization or structure is the self. The self is known to modulate cognitive, somatic, affective, social, and motor changes [5–10] and may therefore be an ideal candidate to provide a more global or basic disturbance (see discussion for definition) of MDD that, in turn, modulates the various functions in an abnormal way. There is indeed strong evidence that the self may be altered in MDD as these subjects show an abnormal focus on their own self in their emotions, cognitions, etc., e.g., increased self-focus [11–14]. Given the assumption that the self is a more global and basic disturbance of MDD [6, 10], one would assume changes in self to be a trait feature (rather than a state feature) which, therefore, should also be present in post-acute MDD [15, 16].

Various brain imaging studies show increased activity in specifically cortical midline structures (CMS) in MDD during self-specific processing [17–22]. What remains unclear, though, is how that affects the regions outside the CMS, namely those regions where affective, cognitive, social and motor functions are processed. A recent study investigated the brain’s global activity (global signal) in fMRI resting state and observed abnormally increased representation of the global signal in specifically the CMS of acute MDD compared to their non-CMS regions [12]. This was complemented by another study showing decreased representation of global brain activity in acute MDD during task-related activity (emotion x speed interaction) in CMS [23]. While these findings hint towards a relationship of altered global brain activity in CMS with the increased self-focus in MDD, they nevertheless leave open (i) the relationship of global brain activity in specifically CMS with the self in MDD; (ii) how that modulates other functions like abnormal emotion regulation [24] in these subjects. Addressing these yet open issues is the goal of our study.

### Aims and hypotheses—connecting the global neural substrates of the increased self-focus to emotion dysregulation

The first aim of our study is to investigate global brain activity of post-acute MDD during an emotion regulation task (see below) in specifically those regions related to the self. For that, we rely on a recent large-scale meta-analysis in healthy subjects showing distinct regions involved interoceptive, exteroceptive, and mental self [7]. Unlike the intero- and exteroceptive self, the mental self is associated with specifically the CMS while recapitulating the regions of both interoceptive (bilateral insula) and exteroceptive (temporo-parietal junction, premotor) self in a nested hierarchical way [7]. Applying this three-layer topography of self for the first time to MDD, we, based on recent findings [11–14], hypothesized that post-acute MDD subjects would show increased global brain activity (global signal representation) in specifically the CMS regions of the mental self (and the exteroceptive self).

The second specific aim was to investigate the behavioral effects of an emotion regulation task that involved both attention and reappraisal of negative and neutral emotions (see [25] for details). Following other findings [26, 27], we hypothesized that post-acute MDD subjects would show decreased emotion regulation resulting in attributing increased emotion severity to especially negative (rather than neutral) emotions.

The third specific aim was to connect the global brain activity (global signal) in the three-layer topography of self with the behavioral measures of the emotion regulation task, e.g., attending and reappraisal). For that purpose, we applied a novel more complex statistical approach that extends beyond simple correlation, namely multinomial logistic regression (MLR) modal. MLR allows us to probe whether the degree of global neural activity changes related to the self, treated as predictor (or independent) variable in the statistical model, modulates the degree of emotion dysregulation considered as outcome (or dependent) variable. We hypothesized that the supposedly increased global brain activity in regions of both mental and exteroceptive self in our post-acute MDD patients modulate their deficient negative emotion regulation. In that case, one would assume the self and its abnormal global brain activity to take on the role as basic disturbance or generative disorder (see discussion for details of these concepts; see also [28]) of emotion dysregulation in MDD.

## Methods

### Subjects

We used fMRI data collected in a previous study [25]. The data set includes 17 subjects who had at least two Major Depressive Episodes (MDD) with >50% HAMD score reduction with pharmacotherapy and did not consult to psychiatrist apart from their routine follow-up (Mentioned as depression group from now on) and their sex/age/education matched control subjects. Subjects’ demographic and medication information are given in Table 1. Maximum of HAMD-17 score of depression group is 17 (Supplementary Fig. 1). Such a unique group was chosen to investigate trait features (their depressive symptoms are not manifest) rather than their state, that is, their acute depressive symptoms are manifest [16, 29, 30]. For taking medication as a covariate, we scored the pharmacotherapy according to previous study [31]. We included medication scores with and without in our main dataset. However, due to non-availability of medication information for half of the subjects, we couldn’t include medication as a covariate for our control datasets. To control for the effect of HAMD on our neural and behavioral target variables, their differences between groups were taken as covariates in fMRI metrics between groups (Supplementary Fig. 2).

**Table 1:** Patients’ demographic features.

	DEP	CTRL	*p* value
AGE	46.68 (3.97)	48.5 (5.46)	*p* = 0.30
EDUCATION	9 (3.54)	7.11 (3.48)	*p* = 0.23
HAMD	12.35 (3.87)	1.46 (1.89)	*p* < 0.001
MEDICATION	4.75 (3.57)	0.22 (0.67)	*p* = 0.001

Values show mean of feature and parenthesis shows standard deviations. Age and Education is matched for groups. HAMD-17 and medication scores differs between two groups. Two-tailed student’s *t*-test is utilized for comparison.

### Task paradigm

**Fig. 1:**
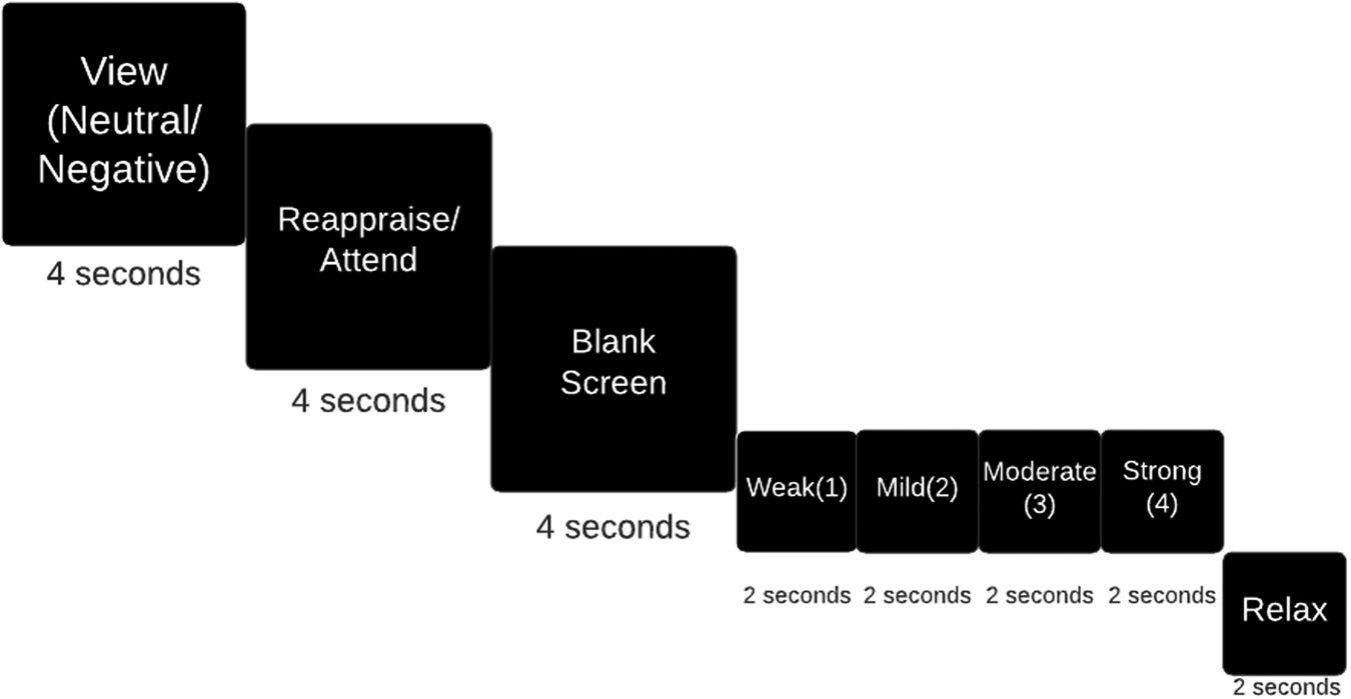
The task paradigm relies on emotional response to negative and neutral images (Simsek et al. [25]). The task consists of 96 trials with 22 s each. Trials are composed of 5 parts. In the first part viewing the image lasts 4 s with “View” displayed on the screen. In the second part, subjects are asked to attend or reappraise to the negative stimuli while only attend to the neutral stimuli. “Attend” or “Reappraise” replaces with “View” for four more seconds. For negative images, “Reappraise” matched 30 pictures while “attend” matched 18 images. In the third part, a black screen appeared on the screen for 4 s. In order to evaluate the success of emotion suppression, subjects rate their emotion severity after the task command with four rating words paired with corresponding numbers (Weak (1), mild (2), moderate (3), strong (4)) with 2 s duration windows for each. Subjects were instructed to press a key with the right thumb located in their right hand. In the fifth part, “Relax” appears on the screen with 2 s duration to prepare subjects for the next trial.

The task paradigm relies on emotional response to negative and neutral images (Fig.1) [25]. The task consists of 96 trials with 22 s each. Trials are composed of 5 parts. In the first part viewing the image lasts 4 s with “View” displayed on the screen. In the second part, subjects are asked to attend or reappraise to the negative stimuli while only attend to the neutral stimuli. “Attend” or “Reappraise” replaces with “View” for four more seconds. For negative images, “Reappraise” matched 30 pictures while “attend” matched 18 images. In the third part, a black screen appeared on the screen for 4 s. In order to evaluate the success of emotion suppression, subjects rate their emotion severity after the task command with four rating words paired with corresponding numbers (Weak (1), mild (2), moderate (3), strong (4)) with 2 s duration windows for each. Subjects were instructed to press a key with the right thumb located in their right hand. In the fifth part, “Relax” appears on the screen with 2 s duration to prepare subjects for the next trial.

### Data acquisition

fMRI images were acquired on a 3.0 Tesla Siemens MAGNETOM Verio MRI scanner with Syngo software and 12-channel Head Matrix coil located at the Department of Neuroradiology, Ege University School of Medicine. High-resolution T1-weighted MP-RAGE gradient-echo anatomical images have the following protocol; 160 × 1 mm sagittal slices, TE: 2.21 ms, TR: 1600 ms, TI: 900 ms, FOV: 256 × 256 mm, image matrix resolution: 256 × 256. Anatomical scans were obtained after the fMRI experiment. T2-weighted Echo-planar imaging (EPI) scans were acquired for the task paradigm. EPI parameters were like following; 23 × 4 mm slices, interleaved from bottom to top (interslice gap: 1 mm, TE: 30 ms, TR: 2000 ms, flip angle: 60°, FOV: 192 × 192 mm, in-plane matrix resolution: 64 × 64, 1056 dynamic scans with 2-s duration).

### Preprocessing

Motion-Corrected BOLD Signal by fMRI (PACE) protocol is used in neuronal data analysis instead of BOLD. The PACE protocol scanner compares 3D volumes acquired in the first and second time-points of the BOLD scan, determines if any motion happened between two time points and changes the position of field-of-view before the third time-point is acquired. It has been shown that using the PACE protocol together with offline head motion correction with regressors associated with less signal loss due to head motion [32]. Preprocessing of fMRI data was completed in Analysis of Functional Neuroimages Software (AFNI). The first two scans were discarded to guarantee proper magnetization. The remaining steps are in the following order; (1) slice-timing correction; (2) despiking; (3) spatial alignment of fMRI data to time frame with a minimum estimated motion; (4) alignment of anatomical data to MNI152 stereotactic space; (5) spatial alignment of fMRI data to skullstripped anatomical data; (6) resampling of voxels (3 × 3 × 3 mm isometric voxels); (7) temporal bandpass filtering between 0.01–0.1 Hz in order to minimize the low-frequency drift and high-frequency physiological noise (respiratory/cardiac noise); (8) Offline head motion correction with twelve regressors (six regressors and its derivatives). In order to check the effect of fast frequency activity in the task paradigm, a data set is produced with only a highpass filter with a lower frequency threshold of 0.01 Hz but without low pass filter. From there on, data sets with and without global signal regression were generated for both traditionally bandpassed (0.01 to 0.1 Hz) and high-passed data (0.01 Hz without low pass), respectively. It has been shown the neuronal source of global signal [33]. Its regression introduces non-existent correlations to fMRI data and loses correlations related physiologically [12, 34–36]. Data sets without global signal regression were utilized as control data set to investigate effect of global signal. Quality control files of afni_proc.py demonstrated head motion above 0.3 mm is below 5% of time points for each subject, thus 17 subjects with past depressive episodes and 14 control subjects included. From there on the traditionally band-passsed data (0.01 to 0.1 Hz) with GS will be mentioned as main data-set where others (0.01 Hz with no low pass) are supplementary.

### Calculation of global signal correlation (GSCORR)

The averaged gray matter BOLD signal is denoted as Global Signal (GS) [37]. Global signal correlation (Z_*GSCORR*_) is the fisher-Z transformation of Pearson correlation’s rho (*ρ*) value between the BOLD signal within voxel/ROI (*x*) and averaged BOLD signal within gray matter mask (*y*) (See eq. 1) [38]. Global Signal Topography is Z_*GSCORR*_ value for each voxel/ROI that measures the contribution of each voxel/region to global brain activity. We calculated GSCORR for each of the three layers of Self. ROI selection for layers explained in the section below, Global Signal Topograhpy- ROI Selection.

1 $$\begin{array}{l}\rho = \underline {n{\sum} {xy} - \left(\sum x\right)\left(\sum y\right)} \\\quad\;\;\; \sqrt{\left[n\sum x^2-(\sum x)^2\right]\left[n\sum y^2-(\sum y)^2\right]} \\ Z_{GSCORR} = \frac{1}{2}\ln \left( {\frac{{1 + \rho }}{{1 - \rho }}} \right)\end{array}$$

This equation is implemented in R 4.1.2 with custom script to calculate GSCORR values.

### Global signal topography—ROI selection

In addition to the predefined regions of the three layers of self [7], we also used a different anatomical template to define the relevant regions in a more independent way by using the well established Glasser Atlas with its predefined 360 regions. Global signal topography (GSCORR) was thus calculated in these purely anatomically defined regions for those regions that are implicated in the self. The regions of the self (interoceptive, exteroceptive, mental) were thus investigated in two ways, one predefined on the basis of their relation to the self using the Qin et al. [7] definitions and once defined independent of their relation to the self by using the Glasser atlas’ anatomical definitions [39]. We calculated GSCORR in both templates for each group, healthy and depressed.

The regions related to the self were selected from a meta-analysis showing self-related brain regions [7]. ROIs were selected according to three layers of self [7], with non-overlapping regions across the three layers of the interoceptive, exteroceptive and mental self (Table 2). This is done to avoid functional overlapping between layers. Regions were picked according to difference in GSCORR with Glasser Parcellation 8 mm radius sphere with each ROI before merging to make a compound ROI for each three-layer with 3dmaskave function of AFNI. For each three layers BOLD signal with PACE protocol averaged to one. The calculation of global signal correlation (GSCORR) is explicated in the next section.

**Table 2:** Selection of Region-of-Interest. ROIs selected from a meta-analysis that shows self-related brain regions (Qin et al. [7]).

ROI	x	y	z
Interoceptive			
Right Insula	34	14	12
Left Insula	−40	−2	2
Exteroceptive			
Right TPJ	−48	−38	36
MPFC	−6	60	22
Mental			
PCC	−4	−54	28
pACC	−6	48	0

ROIs are selected according to three layers of self (Qin et al. [7]). Coordinates are in MNI space. 8 mm radius sphere with each ROI before merging to make a compound ROI for each three layers. For each three-layer PACE-BOLD signal averaged to one. GSCORR is calculated for each layer for both data sets.

### Statistical methods

The codes to replicate statistics, and production of figures can be found at https://github.com/kaanka5312/GSCORR.git. Methods and results can be replicated with the relevant data in the branch. All statistical tests were performed in R 4.1.2 [40]. Wilcoxon tests were corrected with Bonferroni–Holmes method for multiple comparisons. Asterisks implies significance; *p < 0.05, **p < 0.01, ***p < 0.001, ****p < 0.0001.

The two-group whole-brain topography comparison is made relying on the following steps; (1) Wilcoxon test is utilized to compare the two groups’ Glasser regions with a total of 360 regions (180 regions for each hemisphere). (2) In order to control the rate of false positives during multiple comparisons, the two groups’ GSCORR were resampled a hundred times randomly for each Glasser region, and the random *p* value distribution is calculated each time with Wilcoxon test. (3) Expected false discovery rate is set to 0.05. (4) Glasser regions that have a lower percentage of false positives smaller than the threshold, i.e., calculated as false positive smaller than 5% randomly, are considered as significant. This procedure is called positive False Discovery Rate (pFDR) [41].

After showing the topographical difference between the two groups, GSCORR values were compared for each layer of self between groups with the Wilcoxon test due to the number of subjects below 30. Effect of HAMD score differences were accounted for by including them as covariate using ANVOCA. In order to examine the relation of group difference as a categorical variable with behavioral results (i.e., emotion severity), we utilized chi-square test. One subject was excluded from behavioral analysis because she pressed the button after every window during the fMRI task. For multiple answers during rating emotion, first answer is considered.

The effect of continuous neuronal variable GSCORR on behavioral (subjective emotional) results investigated with Multinomial Logistic Regression (MLR). Logistic Regression allows us to investigate the probability of one event referencing another by the linear combination of independent variables (i.e., predictors, ex. Eq. 1). In our model, GSCORR and group variables are predictor variables, and emotion severity is the outcome variable [42, 43]. Since we have four emotion severity states in our task, the test is named as “Multinomial”. To probe the behavioral effects of global signal changes in our datasets, two models were generated, one with GSCORR without GSR and one GSCORR with GSR. On the behavioral side, reference response for logistic regression is considered mild response for negative stimuli and weak response for neutral stimuli because they are the highest number of responses in order to the train model. Next the MLR model was trained and tested with 10-fold cross-validation with 5 repeat. This was done to prevent overfitting of the model to the data by taking 90% of the data for training and 10% data for testing (k = 10). Since selecting folds is based on resampling, this procedure was iterated for 5 times to minimize the randomization effect. Finally, the trained model utilized to predict the emotional responses from three-layer GSCORR and group information by using whole data, with subsequent constitution of a confusion matrix. Statistical analysis and MLR model were completed in R 4.1.2. Data were cleaned with tidyverse [44], statistical analysis operationalized with rstatix [45] and visualized by ggplot2 [46], ggpubr [47] packages and caret package [48] for yielding the MLR models.

## Results

### Neuronal measure—global signal correlation (GSCORR)

#### Qin template—regions predefined as being related to the self

**Fig. 2:**
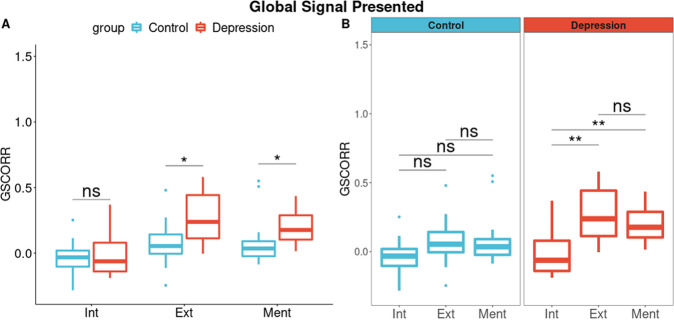
Comparison of GSCORR. **A** In Wilcoxon test, the MDD group shows greater GSCORR in exteroceptive (Mdn = 0.239, *p* < 0.05) and mental (Mdn = 0.177, *p* < 0.05) layers. **B** Three-layer GSCORR compared with each other with the Wilcoxon test. The group with MDD exhibits higher GSCORR in exteroceptive and mental layers compared to GSCORR in the interoceptive layers. No difference exists for the control group. Int = Interoceptive, Ext = Exteroceptive, Ment = Mental. * denotes *p* < 0.05 and ** denotes *p* < 0.01.

We first compared GSCORR values between groups for each of the three layers of self in main dataset. GSCORR in both exteroceptive (*p* = 0.035, *r* = 0.449) and mental (*p* = 0.021, *r* = 0.478) layers was significantly higher in MDD compared to controls (Fig. 2A). No difference in results was obtained when including HAMD scores as covariate. Consistent with Wilcoxon test, mean of GSCORR is significantly greater at Exteroceptive (Control = 0.001 + /−0.056, Depression = 0.307 + /− 0.049, *p* = 0.002) and Mental (Control = 0.021 + /−0.056, Depression = 0.251 + /− 0.049, *p* = 0.030) layers, when HAMD scores are taken as covariate (Supplementary Fig. 2). Accordingly, covariate analysis shows us that group differences in GSCORR scores are not affected by depression severity (HAMD).

We next compared the GSCORR differences between the three layers of self within each of the two groups. Comparing the differences between the layer’s GSCORR (intero-extero, intero-mental, extero-mental), there is no significant difference in the control group. In contrast, the interoceptive GSCORR was significantly lower than both exteroceptive (Mdn = 0.239, *p* = 0.001) and mental layers (Mdn = 0.177, *p* = 0.002) of self in MDD (Fig. 2B). Accordingly, GSCORR between the three self layers is different in the MDD group (*Int* < *Ext*≅*Ment*) but not in the control group (*Ext*≅*Ment*≅*Int*).

Finally, to probe whether these GSCORR changes in the three layers of self are really global (rather than local) changes, we conducted the same analyses with global signal regression. As expected, regression of GS eliminates the group differences for each of the layers as no significant group differences were obtained between groups (Supplementary Fig. 3A, B). Covariate analysis with HAMD scores when GS is regressed exhibits no group difference for any of three layers (Supplementary Fig. 4). Furthermore, application of only highpass filter with GS instead of bandpass filter shows same group difference with main data-set, i.e., higher GSCORR in exteroceptive and mental layers (Supplementary Fig. 5) while no group difference when GS is regressed (Supplementary Fig. 6). When GS is regressed, one can see the GSCORR results down to zero or negative, which is a methodological outcome and shows the succsess of regression procedure (Supplementary Figs. 7, 8).

In sum, results suggest the following; (1) the exteroceptive and mental layer shows greater GSCORR in MDD than in controls which remains independent of HAMD scores. (2) Independence of group difference from HAMD scores further supports our trait features rather than state since each subject HAMD score is below 17. (3) Group difference between MDD and HC is not dependent on low pass filtering and fast frequency fluctuation since band-passing between 0.01–0.1 Hz and only high passing has same significant results; exteroceptive and mental layers have higher GSCORR in MDD group. (4) Exteroceptive and mental layers show greater GSCORR than the interoceptive layer in MDD compared to controls in main data set.

#### Glasser Atlas—whole-brain anatomical definition of regions independent of the self

In order to avoid bias in our selected regions as they were defined in relation to the self (as based on [7]), we also conducted a second series of analyses by using an anatomical definition of the regions independent of their association with the self. For that purpose, we used the standard anatomical template of Glasser (See method). We compared whole-brain topography (GSCORR) with respect to the three layers of self between groups using Wilcoxon test while controlling for false positive results by multiple comparison are with correction using pFDR (see methods). MDD group showed higher GSCORR in Posterior Cingulate Cortex (PCC with subregions POS1, 23d, v23ab, d23ab, RSC, 31pv, 23c, 7 m), Anterior Cingulate and Medial Prefrontal Cortex (ACC and MPFC with subregions p24pr, a24pr, 33pr, p32), Frontoopercular (FOP with subregions FOP2, FOP3, FOP5), temporo-parietal junction (TPJ with subregions SFL and STV) (see Supplementary Fig. 9). In contrast, a lower number of regions shows less GSCORR in MDD like anterior agranular insular cortex (AAIC) (see Supplementary Fig. 12).

Together, these findings highlight increased global signal representation (GSCORR) in the regions of the cortical midline regions in the post-acute MDD group compared to the healthy controls (Supplementary Figs. 9, 11). This further confirms the observation of increased global activity representation in specifically the CMS themselves as possible neural substrate of abnormal self-processing in MDD. When GS is regressed, one can see that the group difference is lost in PCC and the other midline regions—this suggests that the activity increases in MDD in these regions are global rather than local (Supplementary Fig. 10). In contrast, GSCORR is still higher in MDD compared to HC in some other regions including insula and one subregion of ACC/mPFC (p24pr) (Supplementary Fig. 10)—this suggests abnormal local (rather than global) activity in these regions in MDD. Together, these findings support the primarily global rather than local activity increases in CMS of MDD.

Finally, application of the highpass filter (only high pass of 0.01 without any low pass at 0.1 Hz as in the above analyses using the standard bandpass of 0.01 to 0.1 hz) did not change the main result: we again observed higher GSCORR in mainly CMS like PCC, ACC and MPFC in MDD than in HC (Supplementary Fig. 11). Together, these results suggest that narrowing (0.01 to 0.1 Hz) or widening (0.01 Hz without low pass) the targeted frequency range (infra-slow within 0.01 to 0.1 Hz) vs faster of >0.1 Hz) does not change the results: GSCORR increases in mainly CMS regions of MDD were present in both analyses. Albeit tentatively, we, therefore, assume that spatially global activity changes in the CMS (including their association with the mental self) are also global in a temporal way, that is, they seem to occur in a broad-band frequency range holding across the whole frequency spectrum (including infra-slow and slow-fast).

### Behavioral data

**Fig. 3:**
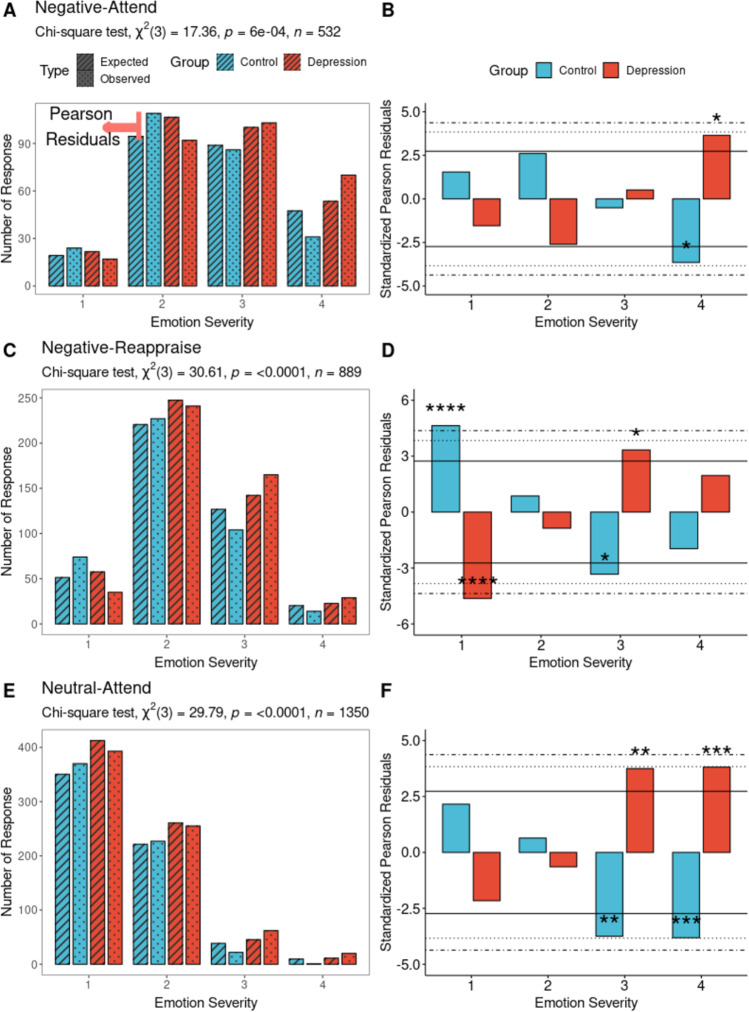
Comparison of emotion severity in reaction to negative and neutral stimuli in MDD and controls. The left column shows emotion severity from 1 to 4 when subjects encounter negative or neutral stimuli. The left column shows the observed number of responses (striped) and expected number of responses (dotted) according to the chi distribution. The Chi-square test’s Z-normalized (standardized) Pearson residuals show significant differences between observed and expected responses. In the right column, standardized Pearson residuals show whether the observed response is significantly different from the expected response according to the chi-square distribution. The horizontal solid line represents the *p* = 0.05 cut-off for z-normal standardized residuals. The horizontal dotted line represents *p* = 0.001, and dot-dashed line represents the *p* = 0.0001 cut-off. **A** During negative stimuli with attending, group difference is related to emotion severity in the Chi-square test. **B** The MDD group shows strong emotional reaction to negative stimuli while attending. **C** During negative stimuli with reappraising, group difference is shown in emotion severity in the Chi-square test. **D** During reappraising the negative stimuli, the control group shows weak emotional response (1), whereas the MDD group exhibits moderate response (3). **E** While attending the neutral stimuli, group difference is shown in emotion severity in the Chi-square test. **F** the MDD group exhibits higher emotional response to neutral stimuli, whereas the control group did not show such pattern. Asterisks implies significance; *p < 0.05, **p < 0.01,***p <0.001, **** *p* < 0.0001.

In the next step, we analyzed the behavioral data. During attention to the negative stimuli (Fig. 3A), the Chi-square test shows significant relation to emotional response severity in the MDD group, $\chi ^2\left( {3,n = 532} \right) = 17.4, p \,<\, 0.001$. Z-normalized (standardized) Pearson residuals of the Chi-square test show significance in the difference between observed and expected responses. That means strong emotional responses (4) do occur often in MDD when these subjects are asked to attend the stimulus whereas no such relation is observed in the control subjects (Fig. 3B).

Similarly, depressed patients show severe emotional responses when they are required to reappraise the negative stimuli (Fig. 3C); e.g., Chi-square test is significant, $\chi ^2\left( {3,n = 889} \right) = 30.61,p \,<\, 0.0001$. Standardized Pearson residuals show that the control subjects exhibit weak emotional responses (1) whereas the depression subjects show moderate emotional responses (3) (Fig. 3D). Finally, when participants attend neutral stimuli, the Chi-square test shows significant relationship with emotional response severity in MDD,$\chi ^2\left( {3,n = 1350} \right) = 29.79,p \,<\, 0.0001$. The depression group shows moderate (3) and strong (4) emotional responses whereas the control group did not.

In sum, results show stronger emotional reaction in MDD subjects than healthy subjects especially in reaction to negative stimuli (Fig. 3B). Given the nature of our paradigm, this suggests failure in emotion suppression during attention and reappraisal (Fig. 3D) to/of negative stimuli. The same applies more or less also to neutral stimuli, which are perceived with the same emotion severity as negative stimuli by the MDD subjects (Fig. 3F).

### Linking neuronal data to behavioral data—Multinomial Logistic Regression

#### Negative stimuli

##### Attending to the emotion

When the global signal is not regressed model for negative-attend, the model accuracy is 47% with *p* = 0.001. Interoceptive, exteroceptive and mental level self GSCORR significantly relate with emotional response severity during emotion attention.

2 $$\ln \left( {\frac{{P\left( {Response = weak} \right)}}{{P\left( {Response = mild} \right)}}} \right) = Intercept + 8.397xGSCORR_{Interoceptive} + \left( { - 2.851} \right)xGSCORR_{Mental}$$

3 $$\ln \left( {\frac{{P\left( {Response = moderate} \right)}}{{P\left( {Response = mild} \right)}}} \right) = Intercept + 2.032xGSCORR_{Interoceptive} + \left( { - 1.418} \right)xGSCORR_{Exteroceptive} + 1.043x\left( {group = DM} \right)$$

4 $${\mathrm{ln}}\left( {\frac{{P\left( {Response = strong} \right)}}{{P\left( {Response = mild} \right)}}} \right) = Intercept + 1.683x\left( {group = DM} \right)$$

In the global signal model, when a weak emotional response is compared to mild response, the relative risk ratio (RRR) for a one-unit increase in the variable interoceptive self GSCORR is 4434.731. In contrast, a one-unit increase in the Mental layer GSCORR is 0.058 (Eq. 2). Moderate emotional response compared to mild and relative risk ratio for a one-unit increase in the variable interoceptive self GSCORR is 7.627, whereas a one-unit increase in the variable exteroceptive self GSCORR is 0.242 (Eq. 3). Having had a depressive episode increases moderate response RRR by 2.839 and strong RRR by 5.382.

**Fig. 4:**
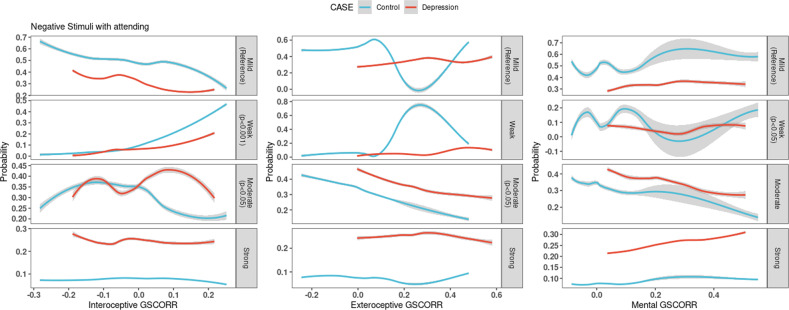
Probability of Emotional response according to GSCORR while attending the negative stimuli. The *x*-axis shows GSCORR value while the *y*-axis shows probability of emotional response relative to the reference. Significant or marginal results are highlighted in gray tabs with respective *p* values. Blue color represents healthy controls while red represents MDD subjects. Increase in interoceptive self GSCORR increases the relative risk ratio of weak and moderate responses compared to mild (First column). When exteroceptive self GSCORR increases, the probability of moderate emotional response decreases compared to mild (second column). When mental self GSCORR increases, the probability of a weak emotional response decreases (third column). Overall, being in MDD group increases the probability of moderate or strong emotional response (as distinguished from mild and weak response).

The results suggest an Increase in interoceptive self GSCORR during weak and moderate emotional reactions, whereas an increase in exteroceptive self GSCORR decreases the chance of moderate emotional reaction in MDD (Fig. 4). Increased mental self GSCORR decreases the chance of weak emotional response. Having had past depressive episodes gradually increases the RRR of emotion severity from weak to strong (Fig. 4).

Finally, we probed whether there is an influence of frequency bands on the association of neural GSCORR with behavioral measures. Removing the low pass by only using a high bandpass of 0.01 Hz which includes the faster frequencies above 0.1 Hz removes all significant GSCORR relationships with behavioral measures yielding only non-significant results (Supplementary Fig. 13). This stands in contrast to the GSCORR results themselves (independent of their association with behavior) where the inclusion of the faster frequency range above 0.1 Hz (in the high bandpass of 0.01 Hz without any low pass) did not change the group differences. Accordingly, inclusion of the faster frequencies (in the high bandpassed data) only affected the relationship of GSCORR and behavior but not the GSCORR group differences themselves. This, albeit tentatively, suggests a special role of the infra-slow frequency range ((0.01 to 0.1 Hz) (as distinct from the faster frequency range >0.1 Hz) for mediating the modulatory impact of increased global neural activity related to the different facets of self (interoceptive, exteroceptive, mental) on the regulation of negative emotion, e.g., emotion attention. Applying a model with GSCORR with GSR (Supplementary Fig. 14), only weak emotional response is related increased interoceptive GSCORR, where we miss relationship between exteroceptive and mental layer with behavior.

##### Reappraising the emotion

In the global signal correlation model for negative reappraisal, model accuracy was 56% with a *p* = 0.022 compared to null hypothesis when there is no model.

5 $$\ln \left( {\frac{{P\left( {Response = weak} \right)}}{{P\left( {Response = mild} \right)}}} \right) = Intercept + 4.378xGSCORR_{Interoceptive} + 2.209xGSCORR_{Exteroceptive} + \left( { - 1.396x\left( {group = DM} \right)} \right)$$

6 $$\ln \left( {\frac{{P\left( {Response = moderate} \right)}}{{P\left( {Response = mild} \right)}}} \right) = Intercept + \left( { - 1.037} \right)xGSCORR_{Exteroceptive} + \left( {0.78x\left( {group = DM} \right)} \right)$$

7 $${\mathrm{ln}} \left( {\frac{{P\left( {Response = strong} \right)}}{{P\left( {Response = mild} \right)}}} \right) = Intercept + 2.778xGSCORR_{Interoceptive} + \left( { - 4.432} \right)xGSCORR_{Exteroceptive} + \left( {1.46x\left( {group = DM} \right)} \right)$$

In the Global signal model for reappraising the negative stimuli, a one-unit increase in the Interoceptive self GSCORR increased weak emotional response relative risk (RRR) by 79.652 compared to mild (Eq. 5). Increase in one-unit Interoceptive self GSCORR increases the RRR of strong emotional reaction by 16.086, marginally (*p* = 0.07, Eq. 7). However a one-unit increase in the Exteroceptive level GSCORR decreases the relative risk of having moderate (0.354, Eq. 6) and strong (0.012, Eq. 7) emotional response compared to mild, whereas it increases the relative risk for weak response by 9.103 (Eq. 5). Having a past MDD episode increases the relative risk of having moderate (RRR = 2.181, Eq. 6) and strong (RRR = 4.307, Eq. 7) emotional response compared to mild, whereas it decreases the relative risk for weak emotional response (RRR = 0.248, Eq. 5). Finally, as in the case of negative emotion attention, relationship of the three layers of the self’s global brain activity with the behavioral measures of negative emotion reappraisal largely disappeared when the high pass filter (0.01 Hz with no low pass filter) was used (Supplementary Fig. 15). This again suggests a special role of the infra-slow frequencies (0.01 to 0.1 Hz) in mediating the modulatory effects of self-based global brain activity on negative emotion regulation, that is, emotion reappraisal.

**Fig. 5:**
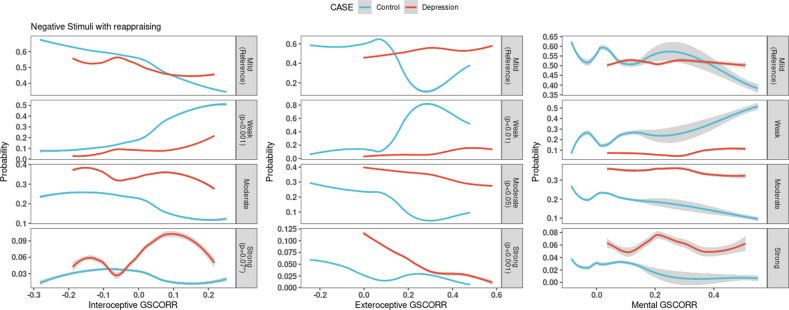
Probability of emotional response according to GSCORR while reappraising the negative stimuli. The *x*-axis shows the GSCORR value while the *y*-axis shows the probability of emotional response relative to the reference. Significant or marginal results are highlighted in gray tabs with their respective *p* values. Blue color represents healthy controls while red represents MDD group. Increase in interoceptive self GSCORR increases the chance of weak emotional response by increasing in strong emotional response marginally (*p* = 0.07, first column). Increase in exteroceptive self GSCORR increases the chance of strong emotional response gradually, from weak to strong (second column). Mental self GSCORR has no significant response (third column). Overall, having a past MDD episode increases the probability of moderate and strong emotional reaction while it decreases the probability of weak emotional response.

In sum, an increase in exteroceptive self GSCORR gradually decreases the chance of a higher emotional response from weak to strong, whereas an increase in interoceptive self GSCORR increases the chance of weak emotional reaction (Fig. 5). In addition, past MDD episodes increase moderate and strong RRR while decreasing weak response probability. Moreover, we again observed a frequency effects with mainly the infra-slow frequencies (0.01 to 0.1 Hz) modulating the relationship of GSCORR and emotion regulation measures. Finally, applying a model with global signal regression, most of the effects disappeared with only strong emotional responses related with a decrease in exteroceptive self GSCORR remaining (Supplementary Fig. 16). Thus, as in the case of emotion attention, GS-behavioral relationship during emotion reappraisal is mostly driven by the global (rather than local) and infra-slow (rather than faster) component of neural activity in the regions related to the three layers of self (interoceptive, exteroceptive, mental).

### Neutral stimuli

The global signal correlation model has an accuracy of 60% with Binomial test being marginally significant (*p* = 0.105) compared to null-hypothesis where no model exists. The global signal correlation model has an accuracy of 59% with a non-significant model (*p* = 0.512). In other words, global signal regression worsens behavioral prediction from neuronal data. Mental and exteroceptive level self GSCORR relate significantly to emotional severity response which disappeared and is eliminated when conducting regression of the global signal (Supplementary Fig. 18).

8 $$\ln \left( {\frac{{P\left( {Response = mild} \right)}}{{P\left( {Response = weak} \right)}}} \right) = Intercept + \left( { - 2.849} \right)xGSCORR_{Exteroceptive} + 1.675xGSCORR_{Mental} + 0.58x\left( {group = DM} \right)$$

9 $$\ln \left( {\frac{{P\left( {Response = moderate} \right)}}{{P\left( {Response = weak} \right)}}} \right) = Intercept + \left( { - 2.02} \right)xGSCORR_{Exteroceptive} + 1.721x\left( {group = DM} \right)$$

In the global signal model, one-unit increase in the exteroceptive self GSCORR decreases the relative risk of having mild (0.058, Eq. 8) and moderate (0.133, Eq. 9) emotional response compared to weak. However, a one-unit increase in the Mental level GSCORR increased mild emotional response relative risk by 5.338 compared to weak. However, increased mental self GSCORR increases weak emotional response probability and decreases mild emotional response probability, while the opposite for past MDD episode group. Having a past major depressive episode increases the relative risk of having mild (RRR = 1.724), moderate (RRR = 5.589) and strong (RRR = 8.145) emotional response compared to weak, gradually.

**Fig. 6:**
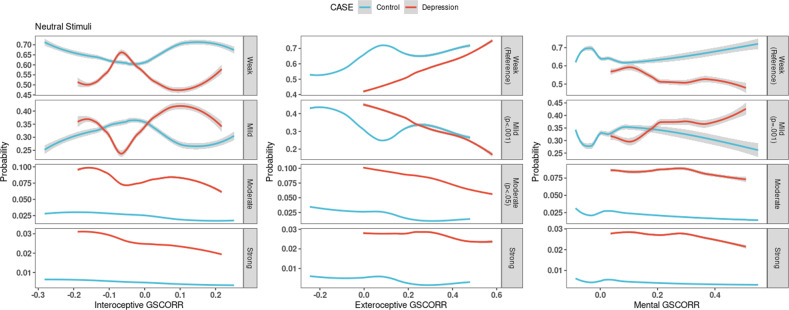
Probability of emotional response according to GSCORR while attending the neutral stimuli. The *x*-axis shows GSCORR value the while *y*-axis shows probability of emotional response relative to the reference. Significant or marginal results are highlighted in gray tabs with respective *p* values. Blue color represents healthy controls while red represents MDD group. Interoceptive self GSCORR has no significant relation with emotional response (first column). Increase in exteroceptive self GSCORR increases the chance of mild emotional response, whereas GSCORR decreases weak emotional response (second column). Increase in mental self GSCORR decreases mild emotional response and increases weak emotional response in the control group (third column). Increase in mental self GSCORR increases the chance of mild emotional response and decreases weak emotional response in the MDD group.

In sum, when attending the neutral stimuli, increased exteroceptive self GSCORR is related to an increased probability of weak response in both groups. While increased self GSCORR in the mental layer increases the probability of weak response in the control group but increases mild response in the MDD group (Fig. 6). Finally, unlike in the negative conditions, application of the high pass filter (0.01 Hz with no low pass) did not affect the results on GSCORR-behavior relationship at all (Supplementary Fig. 17). This suggests a special role of infra-slow frequencies in specifically mediating the relationship of self-based GSCORR with negative emotions (as distinct from neutral emotions).

## Discussion

We here investigated the brain’s global topography of self and its relationship to emotion regulation in post-acute MDD subjects compared to control subjects. Our main findings are: (i) significantly elevated global signal representation (GSCORR) in MDD in specifically those regions mediating the mental and exteroceptive self; (ii) significantly stronger and more severe negative emotional responses during both attention and reappraisal in MDD; (iii) significant relationship of increased GSCORR in specifically mental and exteroceptive layers with increased emotion response severity in MDD. Together, our findings highlight major neural changes in the brain’s global topography of self including how these modulate emotion dysregulation in post-acute MDD. This lends support to the assumption of a basic neural disturbance of self in MDD that, being a trait feature, may exist prior to and independent of the acute depressive symptoms while, at the same time, modulating their underlying emotion dysregulation.

### Increased global signal representation of the mental and exteroceptive self in post-acute MDD

We observed increased global signal representation in specifically the regions of the mental and exteroceptive self like CMS and TPJ using both selected regions related to the self and a more general anatomical template (glasser atlas). This is well consistent with previous studies showing abnormal global brain activity in these regions during both rest [12] and task [23] in acute MDD. Our results confirm the abnormal global activity representation which is further supported by the disappearance of these differences when conducting global signal regression. At the same time, our results extend the previous ones in several aspects.

Firstly, we show that these global signal changes in especially the CMS occur in regions specifically associated with the self, that is, mental and exteroceptive self. Secondly, using a complex model of statistical analyses, namely multinomial regression, we show that these global topographic changes modulate abnormal emotion regulation (see below). Thirdly, we demonstrate these changes in post-acute MDD rather than in acute MDD as in the previous studies on global signal in MDD. This is further supported by (i) that all our MDD subjects showed HAMD-17 scores lower than 17, and (ii) that inclusion of HAMD-17 scores as covariate in our analyses did not change the results at all. Together, this strongly suggests that the here observed neural changes in specifically the exteroceptive and mental self and the psychological findings in emotion regulation including their relationship reflect trait rather than state variables of MDD.

### Global infra-slow neural activity related to the self modulates negative emotion regulation

Our behavioral data confirm previous findings [49, 50] of abnormally increased emotion severity during attention and reappraisal. These findings suggest abnormal cognitive-emotion regulation in MDD which is a well established observation [51, 52]. Importantly, applying a complex statistical model extending beyond simple correlation analyses, namely multinomial regression analyses, we were able to link those behavioral findings to the global brain activity. We observed that the increased emotion severity is related to abnormal modulation of global brain activity representing specifically the mental and exteroceptive self. That suggests a key role of increased global neural activity related to mental and exteroceptive self in modulating emotion regulation including attention and suppression(reappraisal) in MDD. Future studies may want to tighten the link of self and emotion even further by for instance investigating neural variability of the global signal over time, e.g., dynamic neural variability in the regions of the self, in order to connect that to a corresponding timeseries of psychological emotion regulation measures obtained in a dynamic way over time.

Three key observations specify the relationship of self and emotion regulation. First, we demonstrate that removal of the global component in neural activity, when using GSR, also eliminates the finding of a modulatory impact of the three layers of self on the behavioral measures of emotion regulation including both emotion attention and reappraisal (suppression). This suggests a key role of global rather than local activity in modulating the relationship of self and emotion in MDD. Secondly, the relationship of neural measures of self and the behavioral measures of emotion was only observed in the infra-slow frequency domain (0.01 to 0.1 Hz). In contrast, it no longer holds when including the faster frequency in the bandpass (0.01 Hz without low pass) (whereas that was not the case in the purely neural comparison of MDD vs healthy subjects’ GSCORR). Albeit tentatively, this suggests a special role of slower frequencies like the infra-slow (0.01 to 0.1 Hz) in mediating the relationship of self and emotion regulation. Finally, the self-emotion relationship was only observed for negative emotion but not for neutral emotion. Together, this suggests a key role of global infra-slow activity in the exteroceptive and mental layers of self for generating abnormal modulation of negative emotion regulation.

### Basic disturbance of self—from the increased mental self over emotion dysregulation to depressive symptoms

Given that this relationship of global brain activity, self and emotion regulation occurred in post-acute MDD, we, albeit tentatively, assume that an altered topography of self is a trait (rather than state) feature and thus a basic disturbance of MDD. A basic disturbance is a feature that is present even in the non-acute state—it is thus a trait rather than a state feature. However, at the same time, such basic disturbance may, given the “right” context with particular self-specific life events [53, 54], propel or predispose the generation of acute psychopathological symptoms – one may therefore also speak of a generative disorder [28]. The generative aspect of the increased global neural representation of the mental self in MDD may for instance be manifest in its modulation of emotion dysregulation that is known to predispose and underlie the acute depressive symptoms [24]. Moreover, such basic disturbance on the global level of both brain, e.g., global signal representation, and psyche, e.g., self, may account for the co-occurrence of various symptoms domains, e.g., affective, cognitive, motor, sensory, social, in MDD [4]. The idea of self is a basic disturbance of depression converges well with the characterization of the self as basic or fundamental feature that underlies affective, cognitive, sensory, and motor function - this is described as baseline model of self-specificity (BMSS) [6] which states that the self provides a baseline or default for all these functions (see also [55, 56]).

This is well resonant with the concept of “generative disorder” which has been introduced by the earlier psychiatrist Eugen Minkowski in the context of schizophrenia (see [57–59]): “Generative disorder is a pattern of change in the fundamental structure of personality (in the contemporary sense of the term subjectivity), for example, selfhood, spatiality, temporality and relatedness to the world. These structural changes influence the formation of psychopathological manifestations and single symptoms. It is not a disturbance of an isolated cognitive function or mental faculty, but rather a global affection of our presence in the world.” (see [57, 60]). Given our findings, we tentatively suppose that the abnormal shift of global brain activity towards especially the mental self in the three-layer topography of self may be such basic organizing principle: it structures all psychic life by re-balancing the relationship of different functions (cognitive, affective, sensory, motor, social) in an abnormal way as manifest in the co-occurrence of different depressive symptoms [4, 28]. The three-layer topography and its changes are inline with the idea of the narrative self and its self-axis as proposed by Davey [10]. The narrative self is closely related to the mental self and the cortical midline structure in the three-layer topography while the self-axis, in part, overlaps with the regions of the intero- and exteroceptive self in the three layers. Hence, we see strong convergence between Davey’s and our concept of self in both psychological and neural terms.

### Limitations

Some limitations need to be mentioned. Our group is rather small due to the very special nature of our data set. This includes both depression and control group daughters in the original data set; however, due to unbalanced age and education, the daughters were not included in the present analysis. In addition to that, medication scores were unavailable for half of the subjects which limits adding them as a covariate, especially in a low sample size group. Moreover, we did not include a resting state. We would expect the altered topography of self to be present already in the resting state prior to and independent of task-related activity—this remains to be investigated in the future. If so, it would lend further support to our assumption of the three-layer topography of self-serving as a basic disturbance of MDD that as trait feature is already present prior to the outbreak of the acute symptoms. Future studies are thus warranted that directly compare acute and non-acute MDD in both rest and task states.

## Conclusion

In conclusion, we demonstrate increased representation of global brain activity in specifically the regions of the mental and exteroceptive self in post-acute MDD. Moreover, using a complex statistical model, that is, multinomial regression, we show that increased global infra-slow brain activity related to specifically the exteroceptive and mental self modulates emotion dysregulation with increased negative emotion severity. We, therefore, propose that the abnormalities in self, as based on the brain’s three-layer topography, may be a strong candidate for a basic disturbance in MDD: it occurs prior to the acute symptom manifestation while, at the same time, it generates negative emotion dysregulation which, in turn, predisposes subjects to develop acute depressive symptoms.

## Supplementary information

Supplementary Material
