# Supplementary material for: Abnormal global signal topography of self modulates emotion dysregulation in major depressive disorder
Source: Transl Psychiatry. 2023 Apr 3;13:107. doi: 10.1038/s41398-023-02398-2 (PMC10070354; doi:10.1038/s41398-023-02398-2)
Supplement: Supplementary file 1 — Supplementary Material [file 41398_2023_2398_MOESM1_ESM.docx]

Supplementary Material


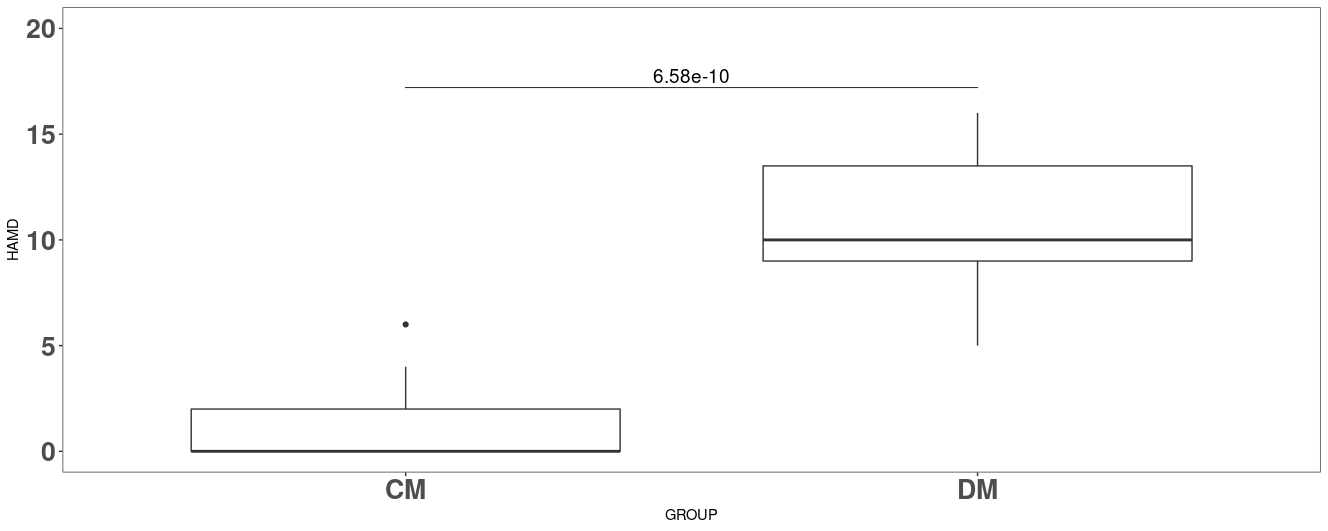


**Supplementary Figure 1***. Comparison of group HAMD scores. Past depressive episode group has higher Hamilton Depression Rating Scale scores (M=11.070) than Control (M=1.385) group.*

# GSCORR comparison between groups and estimated marginal means with covariate

**
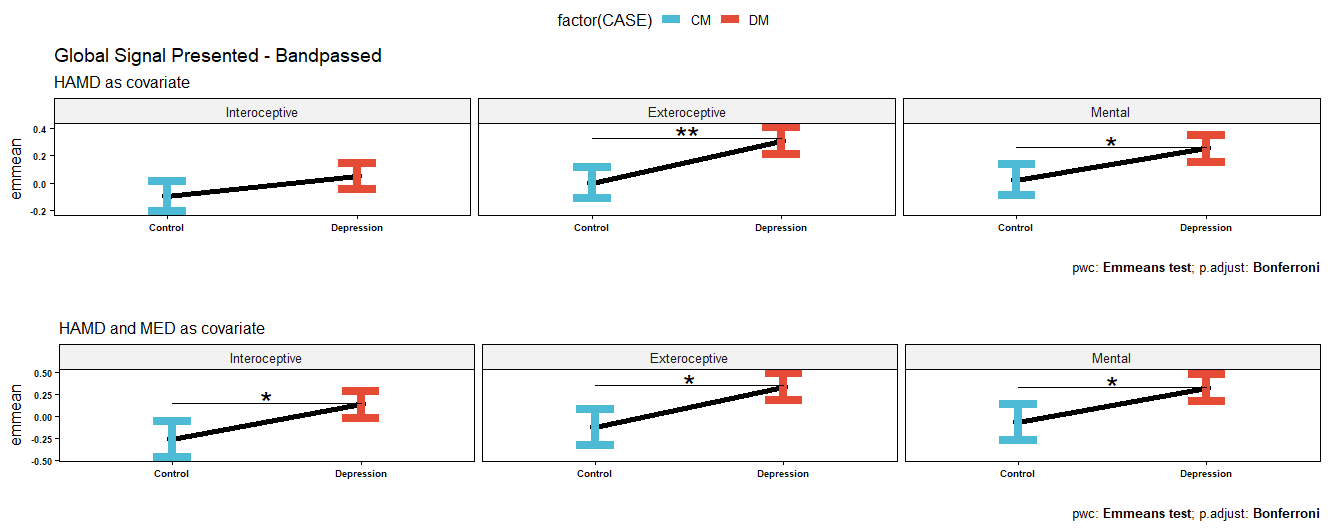
**

**Supplementary Figure 2***.GSCORR difference between groups after HAMD scores are included as covariate. After inclusion of HAMD score difference with ANCOVA, Estimated Marginal Means test (Emmeans test) is used as post-hoc test. p-values are corrected with Bonferoni-Holmes method. Interoceptive layer GSCORR is not statistically significant between groups. In exteroceptive layerpast MDD episodes group showed higher GSCORR (M=0.307,SE=0.049) than control (M=0.001,SE=0.056) group. In mental layer pastMDD episodes group showed higher GSCORR (M=0.251,SE=0.049) than control group (M=0.021,SE=0.056). GSCORR difference between groups after medication is added as covariate with HAMD for the subjects that has medication information, results are consistent for both exteroceptive (Control=-0.060 +/-0.081, Depression= 0.362 +/- 0.081, p=0.015) and mental (Control=-0.042 +/-0.081, Depression= 0.353 +/- 0.081, p=0.025).*

**
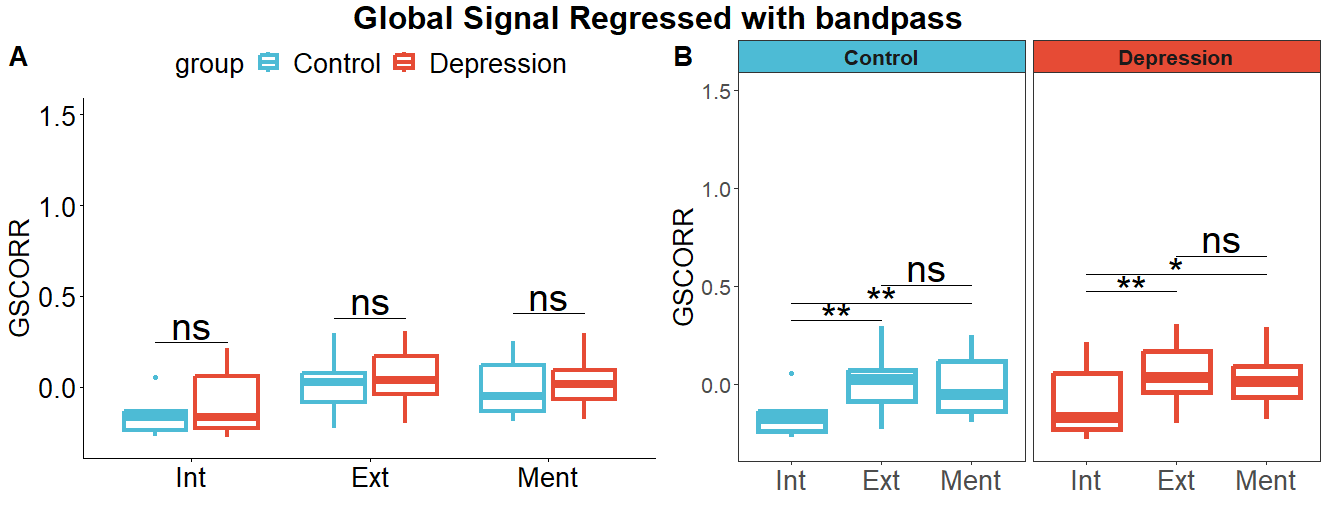
**

**Supplementary Figure 3.** *Layer comparison when global signal regressed when bandpassing between 0.01 Hz to 0.1 Hz applied. Wilcoxon test is used to compare groups due to subject numbers lower than 30. p-values are corrected with Bonferoni-Holmes method. A) No significant difference in Self Layers between groups. B) For the control group exteroceptive layer (Mdn=0.027, SE=0.039) and mental layer (Mdn= -0.05, SE=0.043), GSCORR is higher than the interoceptive layer (Mdn= -0.226, SE=0.037). For the depression group exteroceptive layer (Mdn=0.039, SE=0.036) and mental layer (Mdn= 0.015, SE=0.029), GSCORR is higher than the interoceptive layer (Mdn= -0.227, SE=0.029). Global Signal Topography has a similar hierarchy in two groups (Ment=Ext>Int). Int=Interoceptive, Ext=Exteroceptive, Ment=Mental Layer*

*
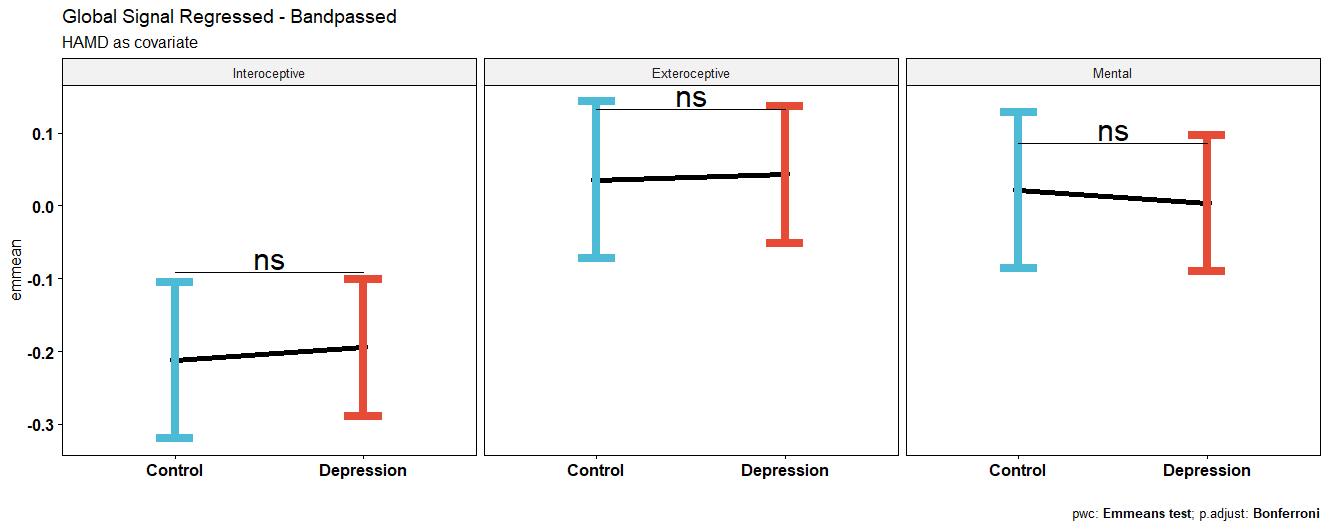
*

**Supplementary Figure 4.** *GSCORR difference between groups after HAMD scores are included as covariate when GS regressed from dataset. It is important to highlight that data is bandpassed between 0.01 Hz to 0.1 Hz. When compared to Supplementary Figure 2, data set shows no difference for any of three layers when GS is regressed. HAMD is controlled as covariate.*

*
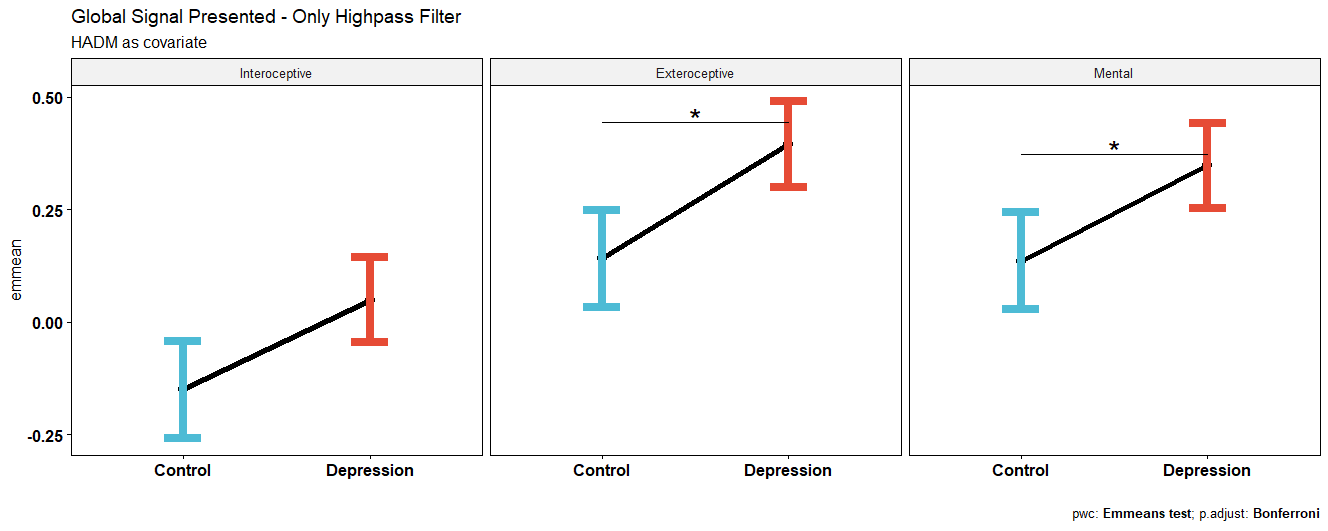
*

**Supplementary Figure 5.** *GSCORR difference between groups when only highpass filter applied over frequencies greater than 0.01 Hz as a control analysis for effect of fast frequency fluctuations. After inclusion of HAMD score difference with ANCOVA, Estimated Marginal Means test (Emmeans test) is used as post-hoc test. p-values are corrected with Bonferoni-Holmes method. Interoceptive layer GSCORR is not statistically significant between groups. In exteroceptive layer MDD group showed higher GSCORR (M=0.395,SE=0.047) than control (M=0.141,SE=0.054) group. In mental layer MDD group showed higher GSCORR (M=0.348,SE=0.047) than control group (M=0.136,SE=0.054). When compared to Supplementary Figure 2, group difference between MDD and HC is not dependent on low pass filtering and fast frequency fluctuation since band-passing between 0.01-0.1 Hz and only high passing has same significant results; exteroceptive and mental layers have higher GSCORR in MDD group.*

*
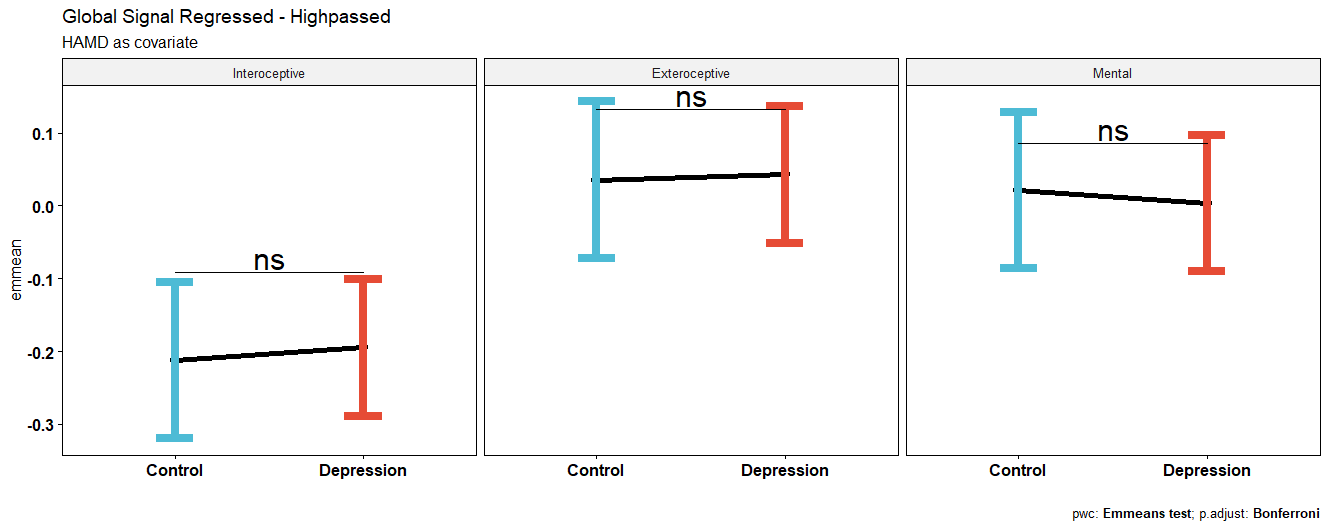
*

**Supplementary Figure 6.** *GSCORR difference between groups after HAMD scores are included as covariate when GS regressed and only highpass filter applied over frequencies greater than 0.1 Hz as a control analysis for effect of fast frequency fluctuations. Global Signal Regression eliminates group difference independent of bandpass or only highpass filtering.*

*
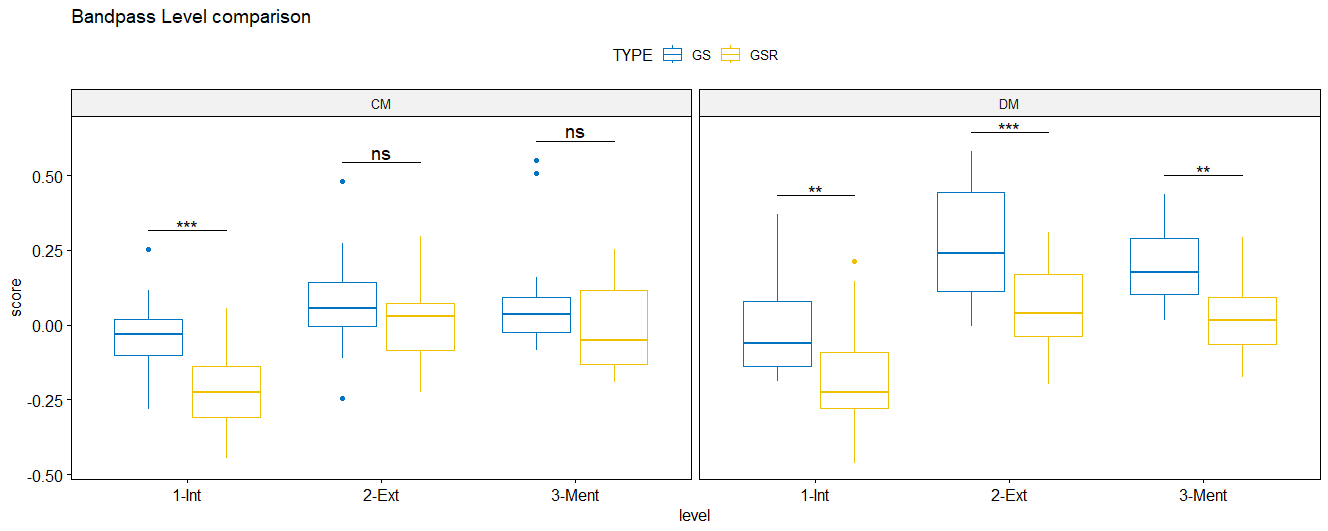
*

**Supplementary Figure 7. GSCORR results with GSR and without GSR.** *Data is bandpassed between 0.01 Hz to 0.1 Hz.* *GSCORR with GSR scores lower than GSCORR without GSR.* *Scores compared with one-way Anova test and post-hoc comparison is performed with emmeans test. In control group, without GSR scores (M_INT_ = -0.027 +/- 0.048 , M_Ext_ = 0.069 +/- 0.048 , M_Ment_= 0.090 +/- 0.048) are statistically higher only in interoceptive layer than with GSR scores (M_INT_ = -0.234 +/- 0.048 , M_Ext_ = 0.013 +/- 0.048 , M_Ment_= 0.000 +/- 0.048). However in depression group shows same statistically higher GSCORR without GSR (M_INT_ = -0.008 +/- 0.038 , M_Ext_ = 0.250 +/- 0.038 , M_Ment_= 0.195 +/- 0.038) than with GSR (M_INT_ = -0.177 +/- 0.038 , M_Ext_ = 0.067 +/- 0.038 , M_Ment_= 0.021 +/- 0.038). Near close 0 scores with GSR is a methodological outcome and shows the succsess of regression procedure. Bandpassing between 0.01 Hz to 0.1 Hz or only highpassing above 0.01 Hz doesn’t effect the success of procedure (Compare with Supplementary Figure 8).*

*
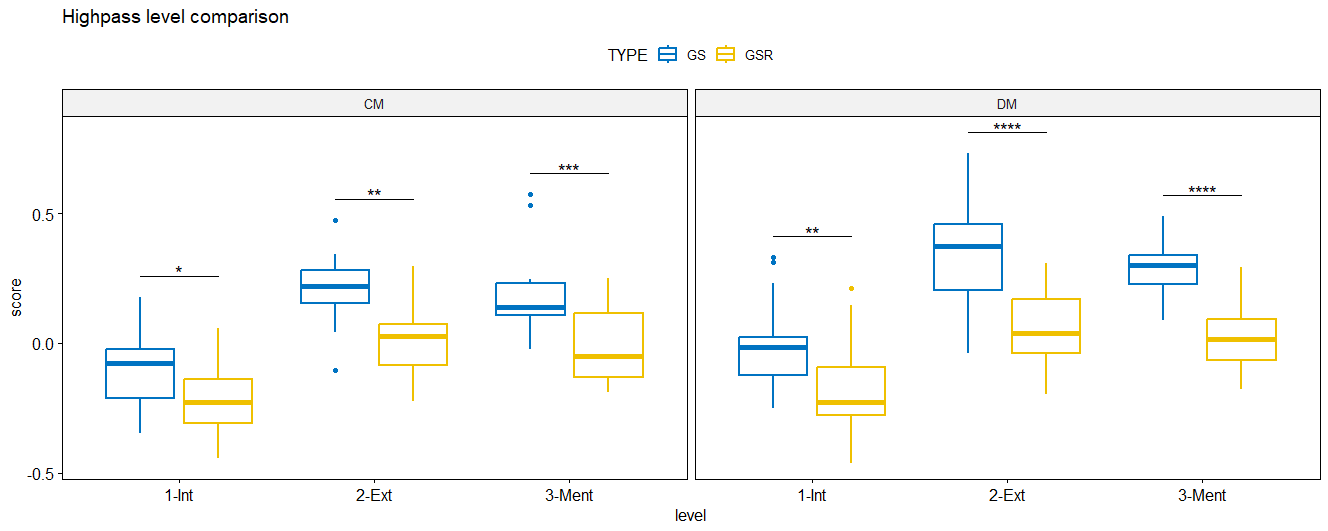
*

**Supplementary Figure 8. GSCORR results with GSR and without GSR.** *Data is only highpass filtered with frequencies greater than 0.01 Hz.* *GSCORR with GSR scores lower than GSCORR without GSR.* *Scores compared with one-way Anova test and post-hoc comparison is performed with emmeans test. In control group, without GSR scores (M_INT_ = -0.086 +/- 0.042 , M_Ext_ = 0.204 +/- 0.042 , M_Ment_= 0.200 +/- 0.042) are statistically higher than with GSR scores (M_INT_ = -0.234 +/- 0.042 , M_Ext_ = 0.013 +/- 0.042 , M_Ment_= 0.000 +/- 0.042) Such as in control group, depression group shows same statistically higher GSCORR without GSR (M_INT_ = -0.003 +/- 0.038 , M_Ext_ = 0.343 +/- 0.038 , M_Ment_= 0.296 +/- 0.038) than with GSR (M_INT_ = -0.177 +/- 0.038 , M_Ext_ = 0.067 +/- 0.038 , M_Ment_= 0.021 +/- 0.038). Near close 0 scores with GSR is a methodological outcome and shows the succsess of regression procedure. Bandpassing between 0.01 Hz to 0.1 Hz or only highpassing above 0.01 Hz doesn’t effect the success of procedure (Compare with supplementary figure 7).*

# Glasser Atlas - Whole brain significant regions

## Post Acute MDD > Control


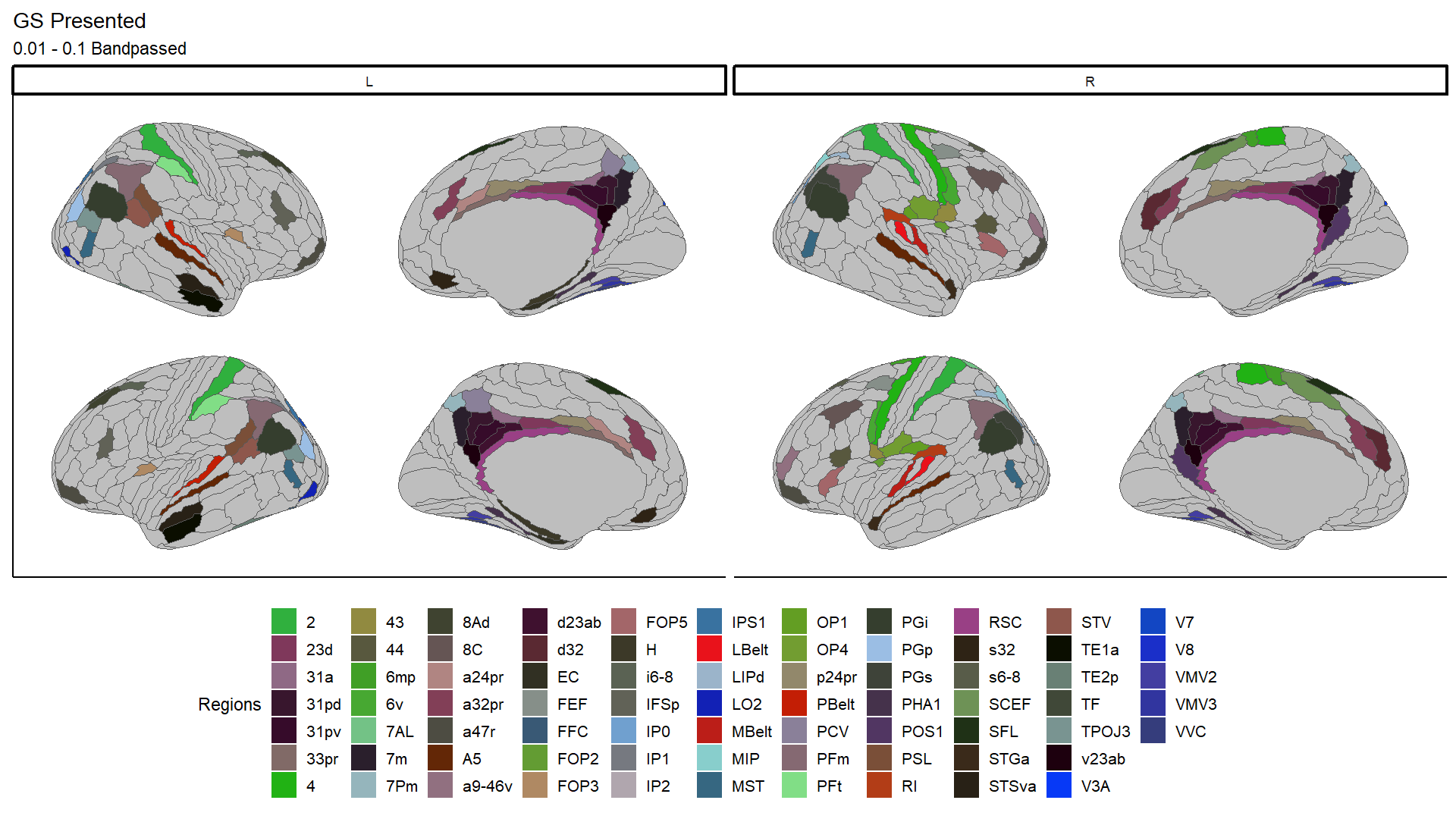


**Supplementary Figure 9.** *Anatomical Atlas (Glasser Atlas) regions that has false positive significance lower than 5% threshold. Multiple comparison results are corrected with pFDR (see Methods). MDD group showed higher GSCORR in Posterior Cingulate Cortex (PCC with subregions POS1, 23d, v23ab, d23ab, RSC, 31pv, 23c, 7m), Anterior Cingulate and Medial Prefrontal Cortex (ACC and MPFC with subregions p24pr, a24pr, 33pr, p32), Frontoopercular (FOP with subregions FOP2, FOP3, FOP5), temporo-parietal junction (TPJ with subregions SFL and STV).*

*
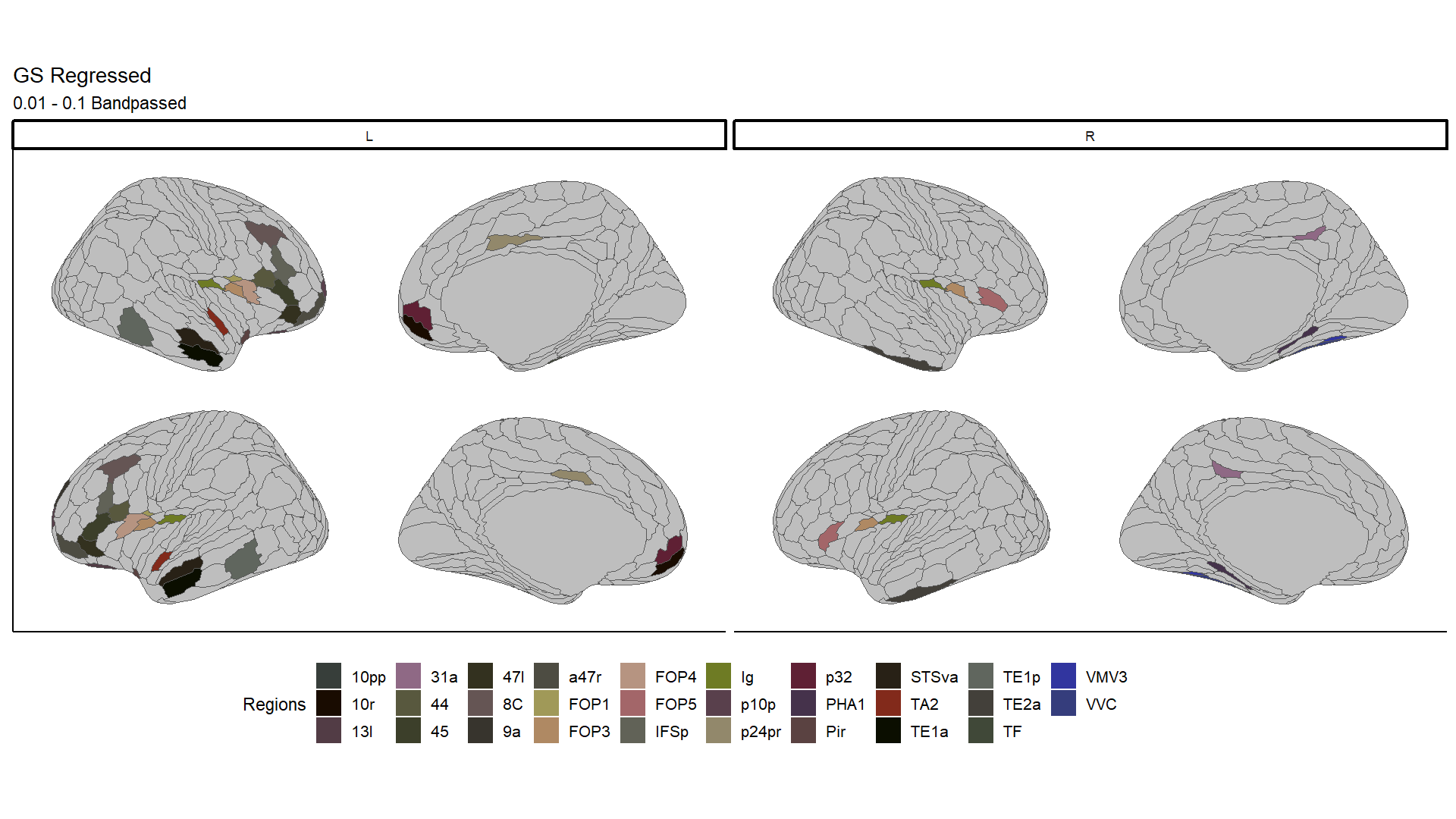
*

**Supplementary Figure 10.** *Anatomical Atlas (Glasser Atlas) regions that has false positive significance lower than 5% threshold. Multiple comparison results are corrected with pFDR (see Methods). When GS is regressed, one can see that the group difference is lost in PCC and the other midline regions – this suggests that the activity increases in MDD in these regions are global rather than local (Compare with Supplementary Figure 9)*.


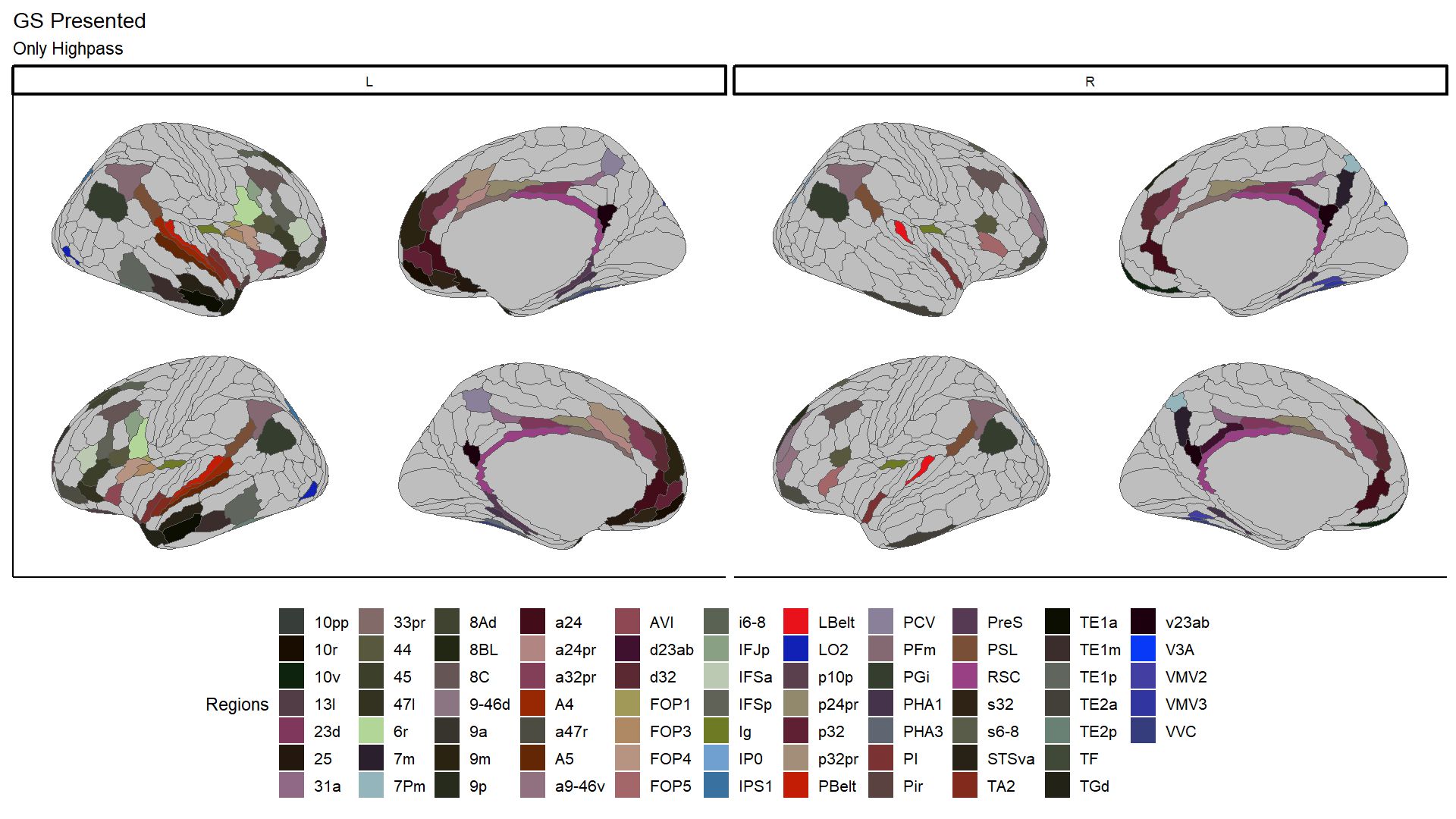


Supplementary Figure 11. *Anatomical Atlas (Glasser Atlas) regions that has false positive significance lower than 5% threshold. Multiple comparison results are corrected with pFDR (see Methods). Application of the highpass filter (only high pass of 0.01 without any low pass at 0.1Hz as in the above analyses using the standard bandpass of 0.01 to 0,1hz) did not change the main result: we again observed higher GSCORR in mainly CMS like PCC, ACC and MPFC in MDD than in HC. Together, these results suggest that narrowing (0.01 to 0.1Hz) or widening (0.01 Hz without low pass) the targeted frequency range (infraslow within 0.01 to 0.1Hz) vs faster of > 0.1Hz) does not change the results: GSCORR increases in mainly CMS regions of MDD were present in both analyses.*

## Control > Post Acute MDD


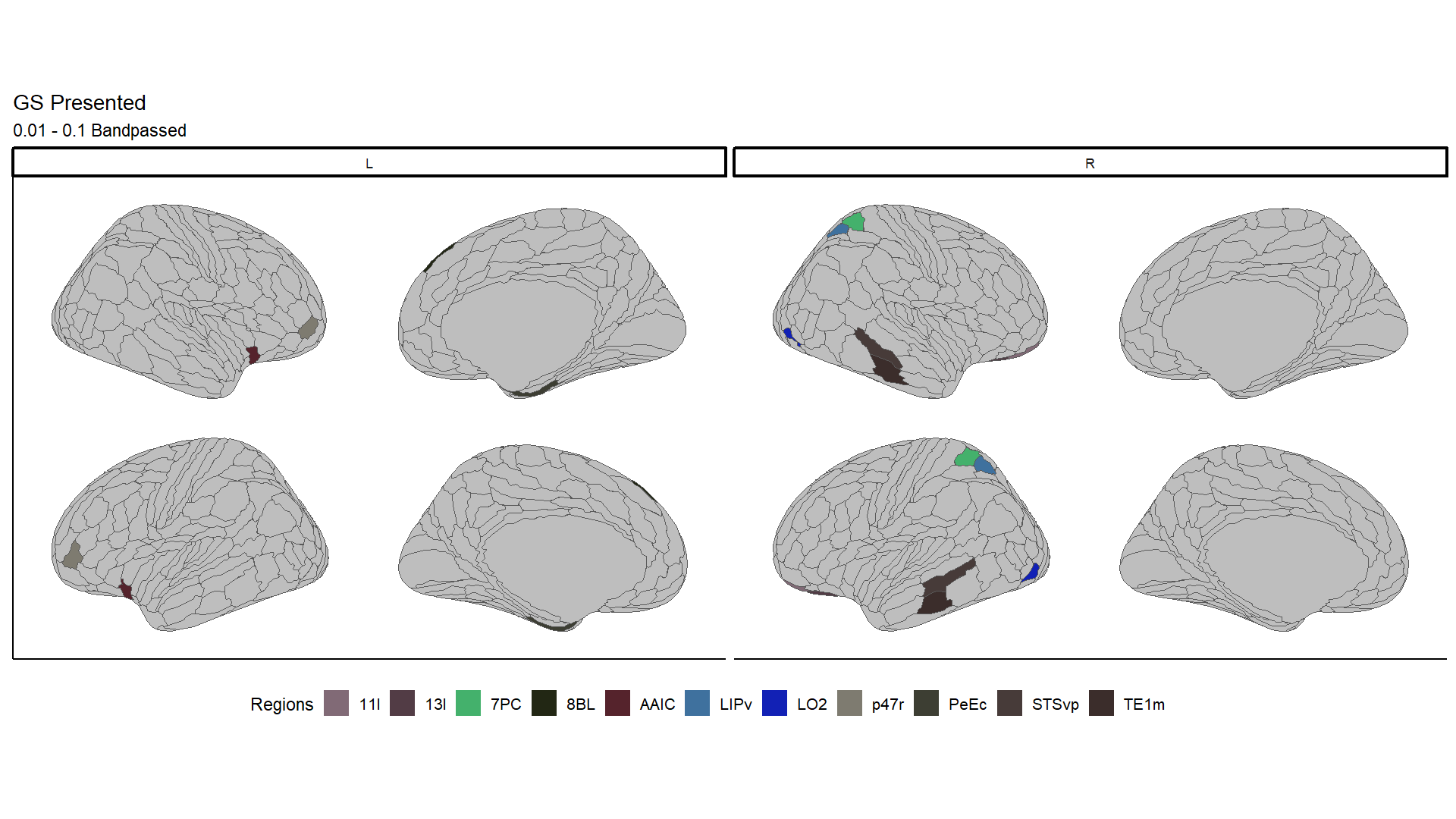


Supplementary Figure 12. *Anatomical Atlas (Glasser Atlas) regions that has false positive significance lower than 5% threshold. Multiple comparison results are corrected with pFDR (see Methods). In contrast, a lower number of regions shows less GSCORR in MDD like anterior agranular insular cortex (AAIC) (Compare with Supplementary Figure 9 ).*

## GSCORR - BEHAVIOURAL DATA RELATION


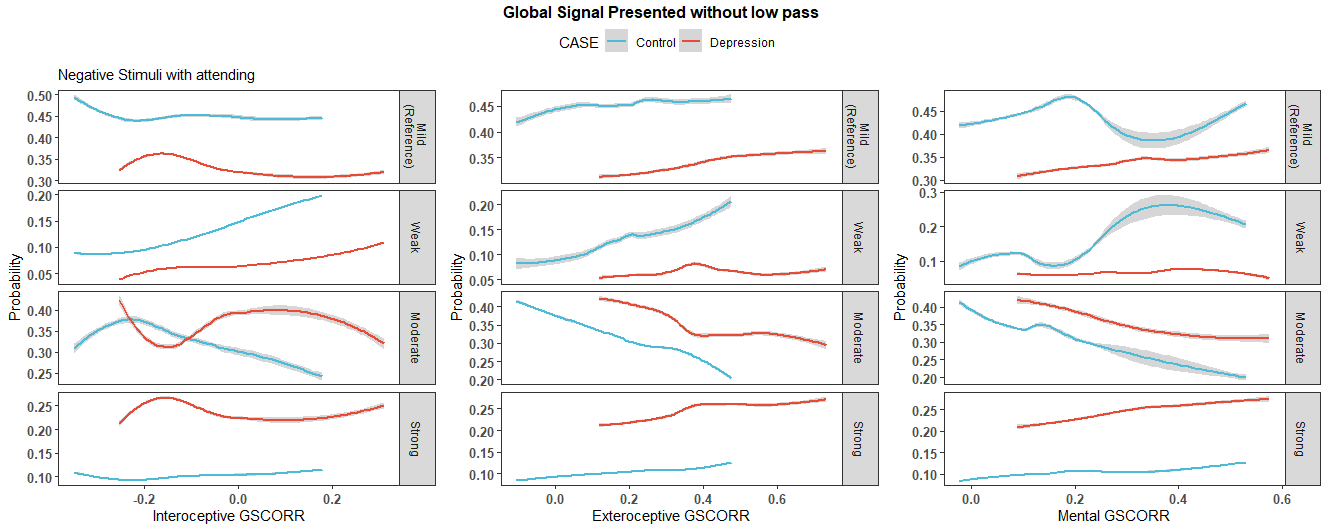


**Supplementary Figure 13.** Influence of frequency bands on the association of neural GSCORR with behavioral measures. Removing the low pass by only using a high bandpass of 0.01Hz which includes the faster frequencies above 0.1Hz removes all significant GSCORR relationships with behavioral measures yielding only non-significant results.


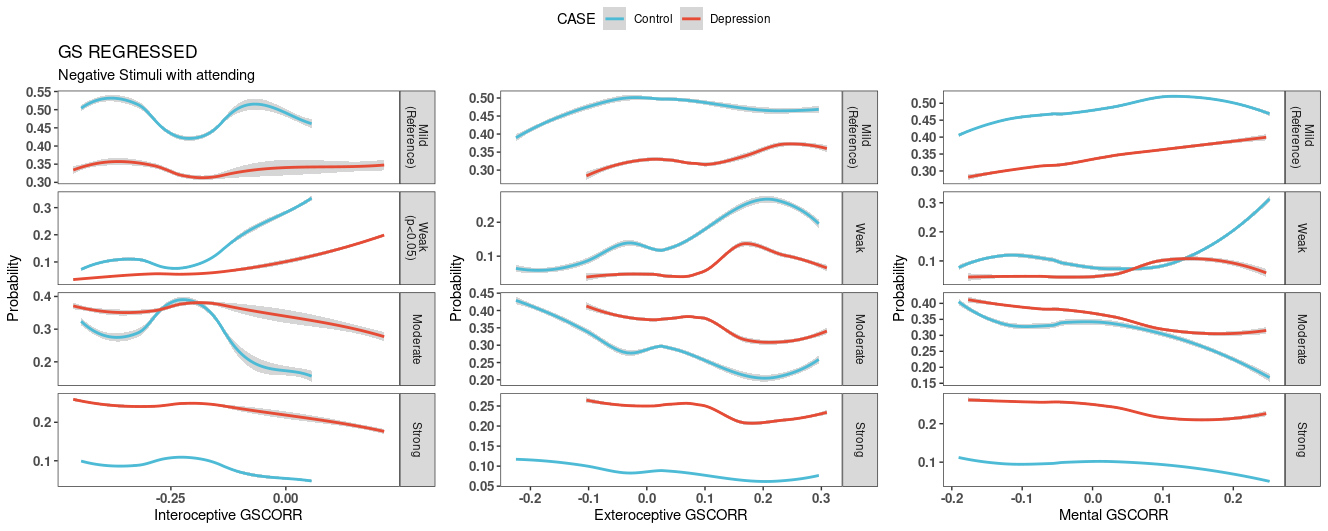


**Supplementary Figure 14***.Negative stimuli while attending while GS is regressed. Only interoceptive layer GSCORR is related with model.*

*
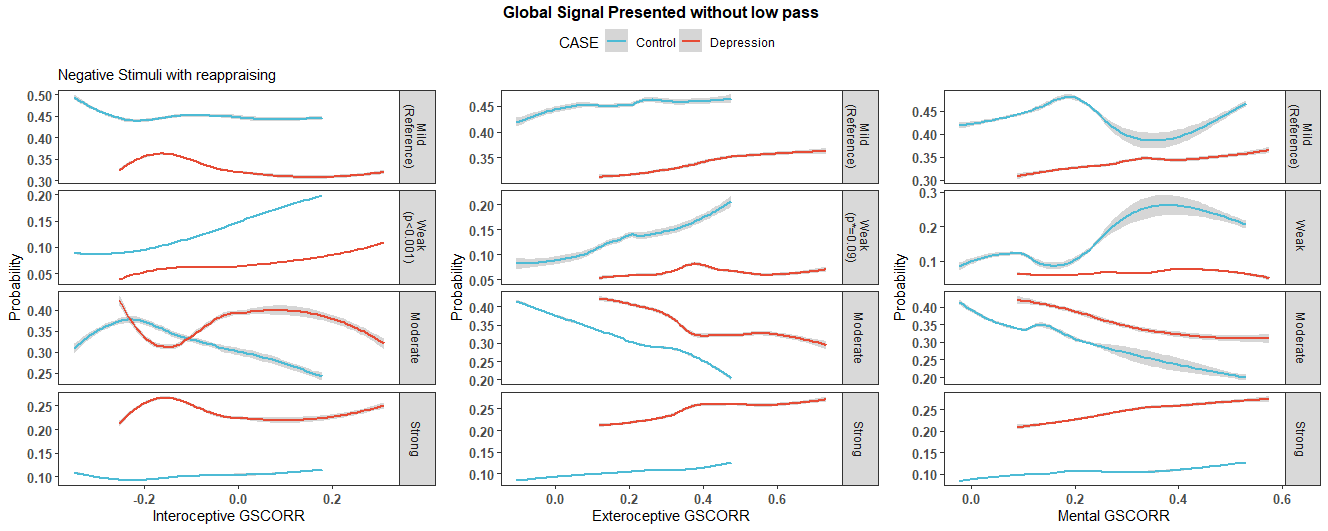
*

**Supplementary Figure 15.** *Relationship of behavioral response with . As in the case of emotion attention, relationship of the three layers of the self’s global brain activity with the behavioral measures of emotion reappraisal largely disappeared when the high pass filter (0.01Hz with no low pass filter) was used. Weak emotional response related with increased interoceptive GSCORR. Increased exteroceptive GSCORR is related with weak emotional response marginally (p=0.09).*


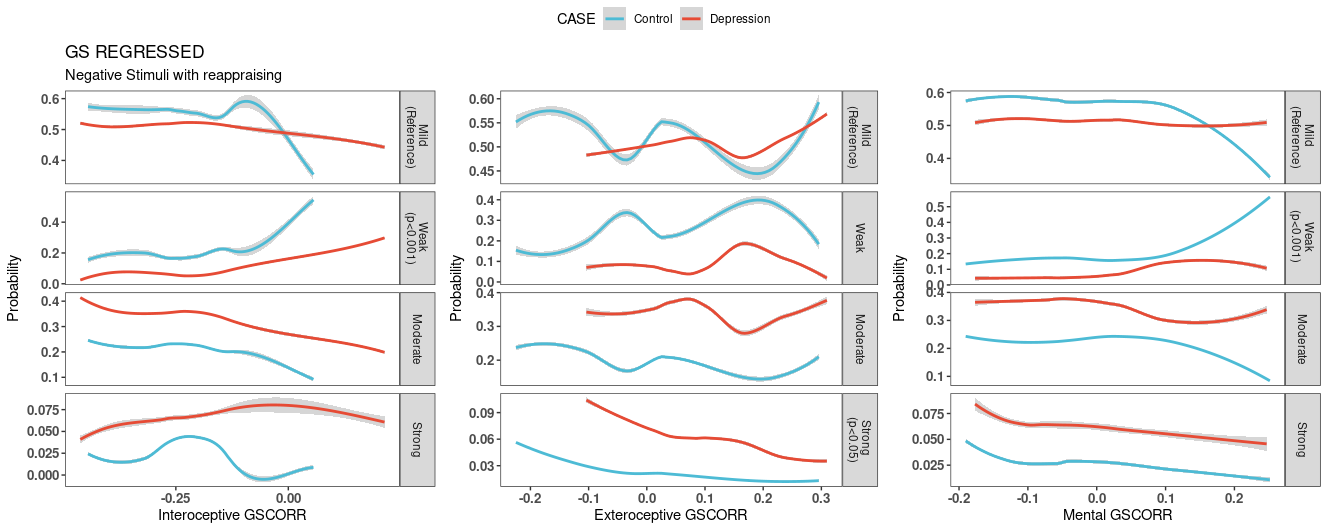


**Supplementary Figure 16***. Negative stimuli while reapprasing while GS is regressed. When compared to GS is not regressed, exteroceptive layer gradual relation from weak to strong is distrupted. Increased weak emotional response is related with increased interoceptive and mental GSCORR. Only strong emotional response is related with increased exteroceptive GSCORR.*

*
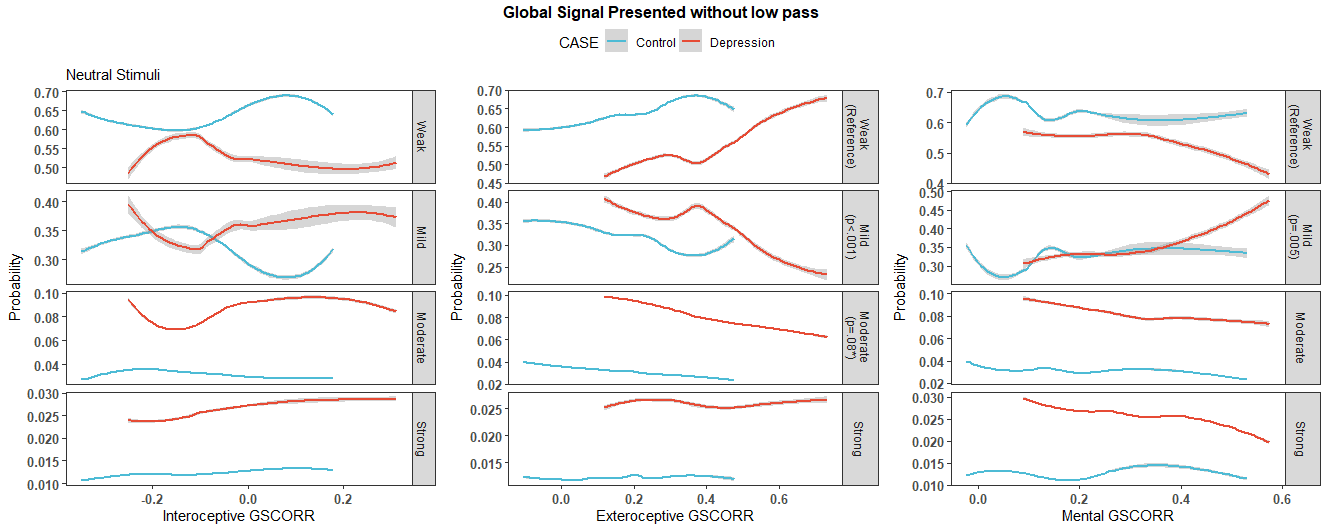
*

**Supplementary Figure 17***. Application of only high pass filter over frequencies 0.01 Hz. Unlike in the negative conditions, application of the high pass filter (0.01Hz with no low pass) did not affect the results on GSCORR-behavior relationship (Compare with figure 6).*


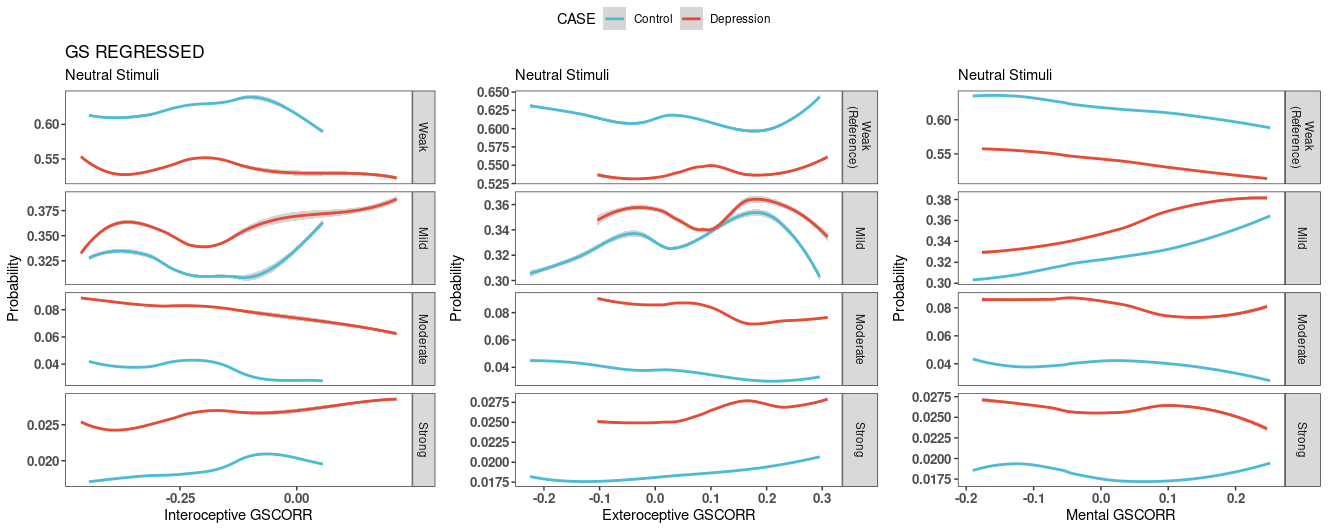


**Supplementary Figure 18***.No significant relation is found between GSCORR and emotion response severity*
